# Enzymatic Characterization and Comparison of Two Steroid Hydroxylases CYP154C3-1 and CYP154C3-2 from *Streptomyces* Species

**DOI:** 10.4014/jmb.2010.10020

**Published:** 2021-01-04

**Authors:** Pradeep Subedi, Ki-Hwa Kim, Young-Soo Hong, Joo-Ho Lee, Tae-Jin Oh

**Affiliations:** 1 Department of Life Science and Biochemical Engineering, Sunmoon University, Asan 31460, Republic of Korea; 2 Chemical Biology Research Center, Korea Research Institute of Bioscience and Biotechnology, Ochang-eup, Chungbuk 28116, Republic of Korea; 3 Genome-Based BioIT Convergence Institute, Asan 31460, Republic of Korea; 4 Department of BT-Convergent Pharmaceutical Engineering, Sunmoon University, Asan 31460, Republic of Korea

**Keywords:** Steroid hydroxylase, *Streptomyces*, hydrogen peroxide, (diacetoxyiodo) benzene, C16 hydroxylation, C–C bond cleavage

## Abstract

Bacterial cytochrome P450 (CYP) enzymes are responsible for the hydroxylation of diverse endogenous substances with a heme molecule used as a cofactor. This study characterized two CYP154C3 proteins from *Streptomyces* sp. W2061 (CYP154C3‐1) and *Streptomyces* sp. KCCM40643 (CYP154C3‐2). The enzymatic activity assays of both CYPs conducted using heterologous redox partners’ putidaredoxin and putidaredoxin reductase showed substrate flexibility with different steroids and exhibited interesting product formation patterns. The enzymatic characterization revealed good activity over a pH range of 7.0 to 7.8 and the optimal temperature range for activity was 30 to 37°C. The major product was the C16-hydroxylated product and the kinetic profiles and patterns of the generated hydroxylated products differed between the two enzymes. Both enzymes showed a higher affinity toward progesterone, with CYP154C3-1 demonstrating slightly higher activity than CYP154C3-2 for most of the substrates. Oxidizing agents (diacetoxyiodo) benzene (PIDA) and hydrogen peroxide (H_2_O_2_) were also utilized to actively support the redox reactions, with optimum conversion achieved at concentrations of 3 mM and 65 mM, respectively. The oxidizing agents affected the product distribution, influencing the type and selectivity of the CYP-catalyzed reaction. Additionally, CYP154C3s also catalyzed the C–C bond cleavage of steroids. Therefore, CYP154C3s may be a good candidate for the production of modified steroids for various biological uses.

## Introduction

The cytochrome P450s (CYPs) are a vast group of heme-containing enzymes found in virtually all living organisms. CYPs have a broad range of substrate specificity and are responsible for the hydroxylation of non-activated carbon atoms, dealkylation, epoxidation, demethylation, sulfoxidation, and carbon-carbon bond cleavage, making them an attractive target as biocatalysts for organic synthesis [1-5]. For catalysis, the majority of the microbial CYPs rely on one or more redox partners to supply redox equivalents. Cytochrome P450 reductases (CPRs) fused to a CYP domain as in the CYP102A and CYP116B family directly deliver electrons to the heme iron while NAD(P)H-dependent ferredoxin reductase (FDR) and ferredoxin (FDX) sequentially deliver electrons to the heme iron from the cofactor [6]. In addition, certain CYPs such as peroxygenases from the CYP152 family use hydrogen peroxide (H_2_O_2_) effectively, without the need for redox partners [7, 8]. Furthermore, some studies have used oxygen surrogates (OSs) to support CYP activity, although lower activity and rapid inactivation of the CYPs limited the efficiency of the OS-supported reactions [9, 10].

Steroids are ubiquitously available bioactive compounds with a wide range of therapeutic effects such as anti-inflammatory, immunosuppressive, anabolic, and diuretic activities [11-14]. Variations in the functional group, type, number, and attachment position to the core steroid subunit directly influence the biological function of steroids. Since steroids are hydrophobic, the presence of the hydroxyl group increases the polarity of the steroid. This may provide a higher level of biological activity or serve as an intermediate for the synthesis of steroidal derivatives. The presence of the hydroxyl group at the C7α position of dehydroepiandrosterone and pregnenolone displayed immunoprotective and immunoregulatory properties. Moreover, it was reported that the C11α-hydroxylation of deoxycortisone was crucial for anti-inflammatory activity, the 17β-hydroxyl function determined androgenic properties, and the 14β-hydroxyl group of steroids was cardio-active [15-19].

In eukaryotes and prokaryotes, steroid hydroxylation is accomplished exclusively by cytochrome P450 monooxygenases [20]. Bacterial monooxygenases compared to those in eukaryotes are advantageous for biotechnological applications because of their solubility and high level of expression [21]. A few CYPs are involved in the hydroxylation of steroids, like the CYP106A family (CYP106A1 and CYP106A2), the CYP109 family (CYP109B1 and CYP109E1), the CYP154C family (CYP154C2, CYP154C3, CYP154C4, CYP154C5, and CYP154C8), and CYP260A1 [17,22-27].

In this study, the cloning, heterologous overexpression, purification, and characterization of two CYP154C3s from *Streptomyces* sp., namely *Streptomyces* sp. W2061 (CYP154C3‐1) and *Streptomyces* sp. KCCM40643 (CYP154C3‐2), were performed. The in vitro reactions of the CYPs with steroids mediated via the NADH-dependent system along with the oxidizing agents (diacetoxyiodo) benzene (PIDA) and H_2_O_2_ were also investigated. Moreover, detailed kinetic and functional characterization of the purified recombinant enzymes were conducted.

## Materials and Methods

### Materials

The steroids used in this study were obtained from TCI (Tokyo Chemical Industry Co., Ltd, Japan). Isopropyl-1-thio-β-D-galactopyranoside (IPTG), 1,4-dithiothreitol (DTT), and kanamycin were purchased from Duchefa Bohemie (Korea). Ampicillin, δ-aminolevulinic acid (ALA), NADH, and formate dehydrogenase were purchased from Sigma-Aldrich (Korea). All of the restriction enzymes, DNA polymerase, T4 DNA ligase, and dNTPs were purchased from Takara Bio (Japan). All other high-grade chemical products were obtained from commercially available sources.

### Bioinformatics Analysis

The nucleotide sequences of CYP154C3-1 and CYP154C3‐2 were deposited in GenBank under accession numbers MF467273 and MT921810, respectively. Identification of the close homologs and comparison of the protein sequences were performed using the Basic Local Alignment Search Tool (BLAST). Multiple sequence alignment was accomplished using GeneDoc [28]. An evolutionary study was conducted using molecular evolutionary genetics analysis (MEGA X) [29]. A phylogenetic tree was constructed using the maximum likelihood method with 1000 bootstrap replicates and the evolutionary distances were computed using the Poisson correction method [30]. The CYP names were assigned by Dr. David Nelson (http://drnelson.utmem.edu/CytochromeP450.html).

### Strains, Media, and Conditions

*Escherichia coli* XL1-Blue (Stratagene, USA), pMD20-T (Takara), pET28a(+), pET32a(+), and pCDFDuet-1 (Novagen, Germany) were used for the subcloning and DNA manipulation. *E. coli* C41 (DE3) (Stratagene) was used as the host cell for recombinant over-expression and whole-cell biotransformation. The *E. coli* strains were grown in Luria-Bertani (LB) media or plates at 37°C supplemented with ampicillin (100 μg/ml), kanamycin (50 μg/ml), and streptomycin (50 μg/ml) as required. X-gal and IPTG were used for colony screening and heterologous protein induction.

### Molecular Cloning and Protein Over-Expression

The CYP154C3-1 and CYP154C3-2 encoding sequences (1,239 bp and 1,254 bp, respectively) were amplified from the genomic DNA of *Streptomyces* sp. W2061 and KCCM40643. The forward and reverse oligonucleotide primers used for polymerase chain reaction (PCR) amplification were 5’- GAA TTC ATG AAC TGC CCG CAC GCC -3’ (EcoRI) and 5’- AAG CTT TCA GCC CAG GAG AAC GGG -3’ (HindIII), respectively, for CYP154C3-1 and 5’- GAA TTC ATG AAC TGC CCG CAC ACT GC -3’ (EcoRI) and 5’- CTC GAG TCA GTC CAG GAG GAC GG -3’ (XhoI), respectively, for CYP154C3-2. The PCR products were cloned into a pMD20-T vector and transformed into *E. coli* XL1-Blue for gene amplification. After confirmation of the sequences, the genes were ligated into the pET32a (+) vector. The resulting construct encoding an N-terminal His6-tag protein was transformed into *E. coli* XL1-Blue and finally into the over-expression *E. coli* C41 (DE3) host. The transformed cells were grown overnight as a seed culture and 1 ml of seed culture was inoculated into 100 ml of LB media supplemented with 100 μg/ml ampicillin and incubated at 37°C (180 rpm). When the cell density reached 0.6 at OD_600_, 1 mM ALA and 0.5 mM FeCl_3_ were added and the culture was further incubated for 20 min at 20°C. After 20 min, the cells were induced with 0.5 mM IPTG and incubated at 20°C for 48 h. The cells were harvested by centrifugation at 3,500 rpm for 20 min and washed twice with 50 mM potassium phosphate buffer (pH 7.4) containing 10% glycerol. The cell pellets were resuspended in 20 ml of the same buffer.

For the in vitro reconstituted system, redox partners putidaredoxin (Pdx) and putidaredoxin reductase (PdR) were over-expressed and His-tagged in *E. coli* BL21(DE3) using plasmid construct pET28a(+) and pET32a(+), respectively, which has been described previously [31].

### Protein Purification and Concentration Determination

The crude extracts obtained by ultra-sonication were centrifuged at 12,500 rpm for 25 min at 4°C to remove cellular debris. The soluble fraction of the cell extracts was mixed with TALON His-tag resin pre-equilibrated with equilibrium buffer (potassium phosphate buffer pH 7.4) and shaken for 60 min. The protein-bound resin was eluted with elution buffers (potassium phosphate buffer pH 7.4 with 10% glycerol) containing 20 mM, 100 mM, and 250 mM imidazole. The fractions containing the proteins of interest were concentrated by ultrafiltration using Amicon centrifugal filters (Millipore) with a 30 kDa cutoff for CYP154C3s and PdR, whereas a 10 kDa cutoff was used for Pdx.

The concentration of the CYP154C3s was measured based on the CO-difference spectra method [32]. Using the potassium phosphate buffer protein, the protein was diluted to 2 ml and separated into two cuvettes (reference and sample). The sample cuvette was bubbled gently with carbon monoxide at a rate of 1 bubble per second for 1 min. Both the reference and sample were reduced with a few grains of sodium dithionite and the spectrum was recorded using a Biochrome Libra S35PC UV/Vis spectrophotometer (England). The concentration of functional CYP154C3s was estimated using an extinction coefficient ε_450-490_ of 91 mm^-1^ cm^-1^ [33]. The PdR concentration was estimated by calculating the average concentration from the 378 nm, 454 nm, and 480 nm wavelengths using extinction coefficients (ε) of 9.7, 10.0, and 8.5 mM^-1^cm^-1^, respectively [34]. The Pdx concentration was estimated by calculating the average concentration from the 415 nm and 454 nm wavelengths using extinction coefficients (ε) of 11.1 and 10.4 mM^-1^cm^-1^, respectively [31].

### Effect of pH, Temperature, and Ionic Strength on Enzymatic Activity

The optimal pH for purified CYP154C3 activity was determined at 30°C using 50 mM potassium phosphate buffer with various pH ranges from 6.0 to 8.5. The maximal enzymatic activity at 30°C using phosphate buffer at pH 7.4 was defined as 100%. For the optimal temperature determination, the enzymatic activity assay was performed at various temperatures from 15–50°C in 50 mM phosphate buffer (pH 7.4). The maximum activity at 30°C in phosphate buffer (pH 7.4) was defined as 100%. The effect of the ionic strength maintained by sodium chloride (NaCl) on CYP154C3 activity was determined by adding different concentrations of NaCl (0–200 mM) into the reaction system. The maximal activity at 30°C in phosphate buffer (pH 7.4) with 70 mM NaCl was defined as 100%. Progesterone was used as the substrate for the characterization of both CYP154C3s in a reaction mixture containing 3 μM CYP154C3s, 100 μM substrate, 6 μM PdR, 24 μM Pdx, 100 μg/ml catalase, and 250 μM NADH. The reaction mixture was extracted twice with ethyl acetate and dried under vacuum for further analysis.

### Enzymatic Activity Assay

**Fig. 1:**
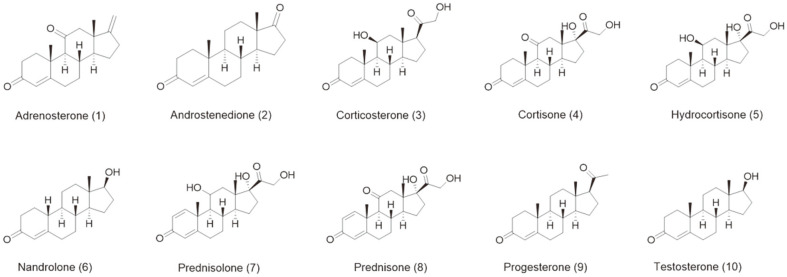
Structure of the steroids used to determine the substrate specificity of the CYP154C3s.

The in vitro assay was carried out in the presence of NADH, H_2_O_2_, and PIDA. Ten substrates were used for the enzymatic activity assays (Fig. 1). The substrates were prepared by dissolving them in dimethyl sulfoxide (DMSO). The reaction mixture contained 3 μM CYP154C3s, 200 μM substrate, 6 μM PdR, 24 μM Pdx, 100 μg/ml catalase, and an NADH-regeneration system comprised of 1 U formate dehydrogenase, 150 mM sodium formate, and 1 mM MgCl_2_ in a final volume of 250 μl in 50 mM potassium phosphate buffer (pH 7.4). The reaction was initiated by the addition of 250 μM NADH and incubated at 30°C for 2 h. The reaction mixture was extracted twice with 250 μl of ethyl acetate dried and analyzed by high-performance liquid chromatography (HPLC). Oxidizing agent (H_2_O_2_ and PIDA)-mediated conversion assays were also performed after determining the optimum concentration required for enzymatic activity. The reaction system consisted of 3 μM CYP154C3s and 200-μM substrate and was initiated by the addition of optimum concentrations of either H_2_O_2_ or PIDA. The reaction was incubated at 30°C for 2 h and extracted as described above.

### Determination of Kinetic Parameters and Catalytic Efficiency

A reaction curve was generated for both the CYP143C3s by measuring the product formed over time from the different substrates. Product quantification was achieved by correlating the peak area of the product(s) with the total peak area of the product(s) and the substrate. The reaction mixture typically contained 1 μM CYP154C3s, 2 μM PdR, 8 μM Pdx, and 100 μM substrate. The reactions were initiated by adding 350 μM NADH. After establishing the initial velocity conditions, the concentrations of all substrates were varied in the range of 0–500 μM to generate a saturation curve. The *V*_max_ and *K*_m_ values of the enzymes for a particular substrate were determined by plotting the rate of product formation versus the corresponding substrate concentration. The kinetic data were analyzed by non-linear regression analysis based on Michaelis-Menten kinetics using the OriginPro program (OriginLab Corporation, USA).

### NADH-Coupling Efficiency

NADH oxidation rates were measured spectrophotometrically using purified CYP, PdR, and Pdx by monitoring the NADH absorbance at 340 nm over time. The reaction mixture typically contained 1 μM CYPs, 2 μM PdR, 10 μM Pdx, and 200 μM substrate in 50 mM phosphate buffer at pH 7.4. The reactions were initiated by the addition of 250 μM NADH (ε = 6.22 mM^-1^ cm^-1^) [31]. The substrate consumption was determined by HPLC. The background NADH consumption in the absence of substrate was also determined. The coupling efficiency was calculated as the percentage of NADH used for product formation over the total NADH consumption.

### Determination of the Substrate Dissociation Constant

Substrate binding assays were performed by the spectrophotometric titration of the enzymes (CYP154C3s) in 50 mM potassium phosphate buffer (pH 7.4) with increasing substrate concentrations until saturation. The absorbance spectra of all samples were recorded from 350 to 500 nm using a Biochrome Libra S35PC UV/Vis Spectrophotometer (England). The *K*_d_ value was calculated by plotting the difference in absorbance (Abs_390_-Abs_420_) against the substrate concentration. The titration data points were fitted to a nonlinear tight-binding quadratic equation [35] (Equation 1) using OriginPro to determine the K_D_ values.

$$A_{\mathrm{obs}}=A_{\max}(([S]+[E_{t}]+K_{D})-({\left([S]+[E_{t}]+K_{D}\right)}^{2}-{\left(4[S][E_{t}]\right)}^{0.5})/2[E_{t}]$$ (1)

In Eq. (1), A_obs_ is the absorption shift determined at any ligand concentration, A_max_ is the maximal absorption shift obtained at ligand saturation, [E_t_] is the enzyme concentration used, [S] is the substrate concentration, and K_D_ is the apparent dissociation constant for the enzyme–ligand complex.

### HPLC and LC-MS Analysis

After drying, the extracted reaction mixture was used for analysis. The dried residue was dissolved in HPLC-grade methanol, filtered through a 0.45‐μm pore polytetrafluoroethylene filter, and analyzed by ultra-high-performance liquid chromatography (UHPLC). The sample was injected and separated using a Mightysil reverse phase C18 column (4.6 × 250 mm, 5 μm). Acetonitrile (B) and water (A) were used as the mobile phase in a gradient system of B at 15% for 0–10 min, 50% for 10–20 min, 70% for 20–25 min, and 15% for 25–40 min at a flow rate of 1 ml/min. Detection of the substrates and their product was performed by UV absorbance at 242 and 245 nm. Liquid chromatography-mass spectroscopy (LC-MS) analysis was performed with a SYNAPT G2-S/ACUITY UPLC liquid chromatography quadrupole time-of-flight/electrospray ionization mass spectrometer (Waters, USA) in the positive ion mode. The products formed were identified by comparison to products reported previously [17, 25, 36].

## Results and Discussion

### Bioinformatics Analysis

**Fig. 2:**
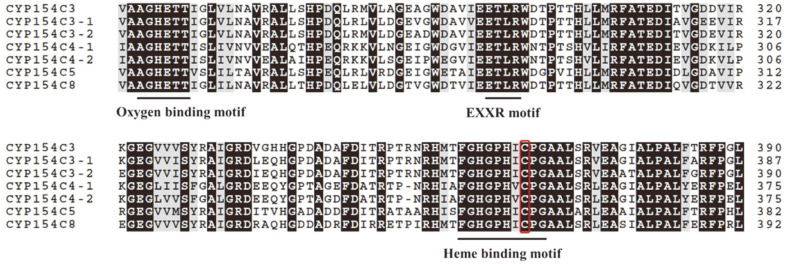
Multiple sequence alignment of *Streptomyces* CYP154Cs with CYP154C3, CYP154C4, and CYP154C5. The conserved and similar residues are highlighted. The highly conserved, functionally relevant regions (oxygen binding motif, EXXR motif, and heme‐binding signature motif) are underlined. The conserved cysteine residue of heme-binding motif is highlighted in red.

**Fig. 3:**
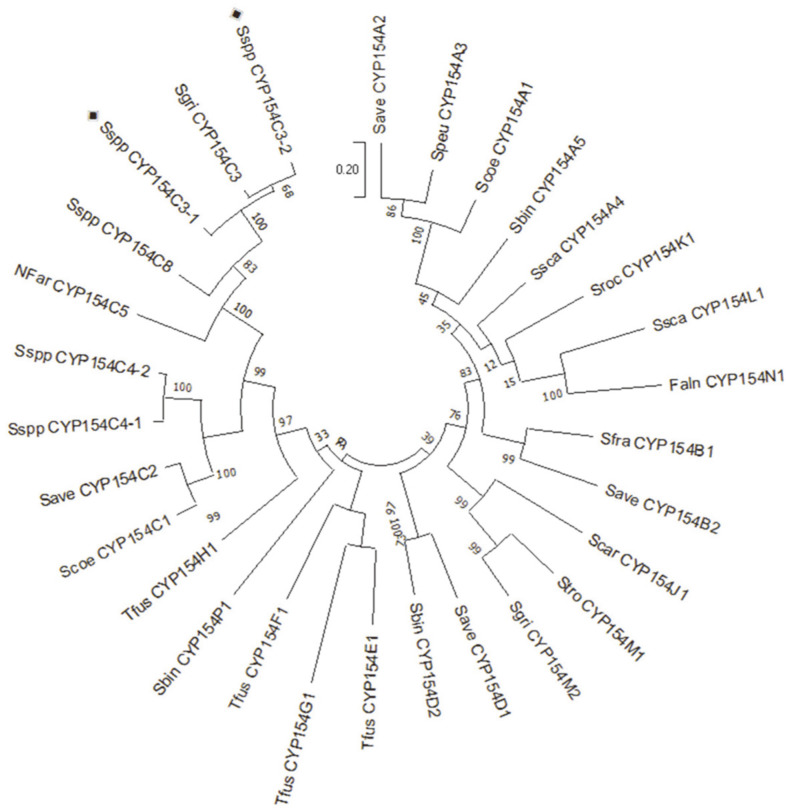
Phylogenetic tree of *Streptomyces* CYP154C3s and their closest homologs. The phylogenetic tree was based on amino acid sequences of the CYP154 family. The phylogenetic tree was constructed by the maximum likelihood method and the Poisson correction model. The prefixes in front of the CYP number abbreviate the genus and the species of the respective bacteria from where the enzyme originated (Faln, *Frankia alni*; Nfar, *Nocardia Farcinia*; Save, *Streptomyces avermitilis*; Sbin, *Streptomyces bingchengensis*; Scar, *Streptomyces carzinostaticus*; Scoe, *Streptomyces coelicolor*; Sfra, *Streptomyces fradiae*; Sgri, *Streptomyces griseus*; Speu, *Streptomyces peucetius*; Sroc, *Streptomyces rochei*; Ssca, *Streptomyces scabies*; Sspp, *Streptomyces* sp.; Stro, *Salinispora tropica*; and Tfus, *Thermobifida fusca*). The P450s in the current study are indicated by the symbol (◈). The vertical bar in the tree represents 0.2 amino acid substitutions per amino acid for the branch length.

Multiple sequence alignment of the selected proteins was performed to observe sequence conservation and the presence of the signature motif. The characteristic conserved oxygen-binding and activation I-helix motif, K helix (EXXR) motif, and heme-binding domain for the CYP family were observed (Fig. 2). All proteins contained an acid-alcohol pair, glutamate, and a threonine residue, which facilitates oxygen activation in CYPs [37]. Homologs of the protein sequences were searched by conducting a PSI-BLAST search (NCBI server). A phylogenetic tree was constructed using the protein sequences of the CYP154C3s and their closest homologs (Fig. 3). Phylogenetic analysis revealed that the CYP154C3s clustered closer to the previously studied *S. griseus* CYP154C3 and *Streptomyces* CYP154C8. Moreover, protein sequence alignment showed that CYP154C3-1 and CYP154C3-2 shared 92.7% and 91.3% identity with CYP154C3, and 75.8% and 76.4% identity with CYP154C8, respectively. These CYPs have been reported to hydroxylate steroids at the C16α-position [17, 24].

### Cloning, Overexpression, Purification, and Spectral Characterization of Proteins

The DNA fragments encoding the CYP154C3s genes were PCR-amplified and cloned into the pET32a (+) expression vector. Both CYPs, CYP154C3‐1 and CYP154C3‐2, showed a better co-expression of target proteins in the soluble form in *E. coli* BL21 (DE3) cells. Soluble protein bound to resin was eluted with a potassium phosphate buffer with different concentrations of imidazole (20 mM, 100 mM, and 250 mM). Eluted fractions, using different concentrations of imidazole, were confirmed by SDS-PAGE analysis. 100 mM imidazole-eluted proteins were obtained at the higher molecular weight (~65 kDa) (Fig. S1A), although the theoretical molecular mass calculated for both CYPs was ~45 kDa. The difference in molecular masses was due to the Trx‐His‐s‐enterokinase fusion sequence in the pET32a(+) vector, which is transcribed and translated along with the CYP154C3s sequence.

The cytosolic purified fraction of the CYP154C3s showed spectral properties characteristic of CYP enzymes by UV–Vis absorption spectroscopy. The carbon monoxide-bound, dithionite-reduced form of the CYP154C3s exhibited absorption maxima at 449 nm, the characteristic signature of CYP heme in its Fe^2+^CO complex form [38].

### Substrate-Binding Assay

The binding of the substrates to the active site of CYP was observed by the displacement of the heme water ligand (the sixth ligand to heme iron). This resulted in a shift of the ferric heme iron from a low-spin to a high-spin state (so-called type I shift) with a minimum Soret absorption of around 420 nm and a maximum of around 390 nm [39, 40]. All of the steroids were tested for a possible type I spin shift in the CYP154C3s. Upon binding to CYP154C3s, the steroids exhibited a type I shift with a maximum absorbance at 390 nm and a minimum at 420 nm (Fig. S1). By titrating different concentrations of the substrates until saturation and fitting to a nonlinear tight-binding quadratic equation, the dissociation constant (*K*_d_) values for the CYP154C3s were determined (Table 1)(Fig. S2). The binding of the steroidal substrate to CYP154C3-1 was tight, with dissociation constants (*K*_d_) lower than those for CYP154C3-2. The dissociation constant (*K*_d_) values for **1**, **2**, and **9** were found to be lower than 0.5 μM, indicating tighter binding. These substrates were the most hydrophobic used in the experiment. In contrast, 5 and 7 were the least hydrophobic and showed high *K*_d_ values. It was observed that decreases in the hydrophobicity of the substrate increased the *K*_d_ value significantly. As reported previously, CYP154C3, CYP154C4, CYP154C5, and CYP154C8 also displayed tight-binding (low *K*_d_) to steroids [17, 24, 25, 41]. Binding of hydrophobic steroids to the active site led to the release of the low-entropic water molecule from the solvation shell of the steroids, subsequently increasing the overall entropy of the system. This resulted in large hydrophobic contact surfaces, which promoted steroid binding to the active site of the enzyme [41].

### Effect of pH, Temperature, and Ionic Strength on Enzymatic Activity

**Fig. 4:**
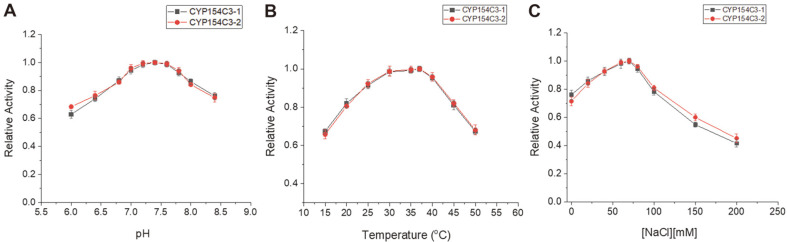
Optimum pH and temperature and the effect of ionic strength on CYP154C3-1 (■) and CYP154C3-2 (●). (**A**) Optimum pH. The CYP154C3-1 and CYP154C3-2 activities were measured over a pH range of 6.0–8.5 at 30°C for 1 h. Both the CYPs favored alkaline pH (7.0–7.8), retaining more than 92% of the maximal activity. (**B**) Optimum temperature. The activity of reactions at 15–50°C in pH 7.4 was measured for 1 h. At a 30–37°C temperature, the enzyme retained more than 98% of maximal activity. (**C**) The effect of ionic strength on the catalytic activity of the enzymes in various ionic strength solutions maintained by NaCl (10–200 mM). A bell-shaped curve revealed improvement in activity in lower ionic strength, while higher ionic strength (>70 mM) decrease the enzyme activity. The hydroxylation of progesterone was measured. The values are the mean of three independent experiments with standard deviation.

The catalytic activity of an enzyme is highly dependent upon assay conditions such as pH, temperature, and ionic strength. The maximal activity of the purified CYP154C3s was observed in potassium phosphate buffer at pH 7.4. Both CYP154C3s showed good pH stability over the pH range of 7.0–7.8, retaining more than 92% of the maximal activity (Fig. 4A). The activity performance as a function of temperature was also very similar for both CYP154C3s. The optimal temperature range for both CYP154C3 activities was 30–37°C, retaining more than 98%of the maximal activity (Fig. 4B). The activity started to decrease dramatically at temperatures above 40°C. Since there is a strong electrostatic interaction between CYP and its redox partners, which is strongly based on charge pair interactions in addition to hydrophobic interactions [42, 43], the ionic strength dependency of the electron transfer from the redox partners to the CYPs was investigated. The catalytic activities of CYP154C3s in various ionic strength conditions maintained by NaCl (10–200 mM) were analyzed (Fig. 4C). The activity of both enzymes showed a marked bell-shaped dependence upon ionic strength. Lower ionic strength improved the enzymatic activity, reaching a maximum at 70 mM for both enzymes. Elevating the ionic strength above 70 mM decreased the enzymatic activity. Higher ionic strength disrupted the electrostatic interactions of the CYP redox partner complex, thereby decreasing the activity [44, 45].

### Enzyme Kinetic Studies

**Fig. 5:**
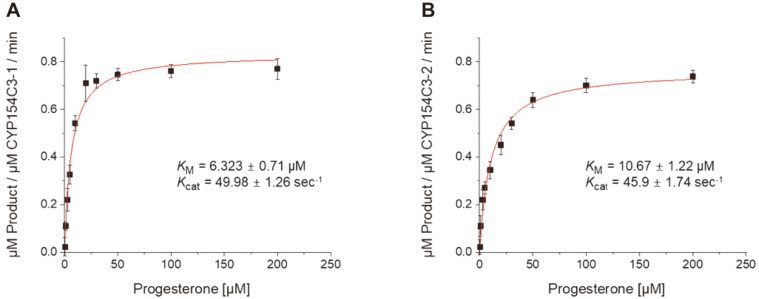
Hyperbolic fit for progesterone. The reaction was catalyzed by CYP154C3-1 (**A**) and CYP154C3-2 (**B**) using Pdx-Pdr-NADH. The reaction mixture contained CYP: Pdx: Pdr at a ratio of 1: 8: 2 with varied substrate concentration and the reaction was started by the addition of 250 μM NADH. The graph of rate of reaction vs substrate concentration was plotted. Data were analyzed by non-linear regression analysis based on Michaelis-Menten kinetics. The values are the mean of three independent experiments with standard deviation.

To gain a deeper understanding of the enzymes, the overall kinetic parameters of purified CYP154C3s were evaluated by directly analyzing the product formation from a panel of substrates by HPLC. The measurements were carried out using purified CYP154C3s in the presence of the heterologous redox partners Pdx and PdR from *P. putida* since the natural redox-partners of CYP154C3s are not known. The Michaelis-Menten constant (*K*_m_) and the catalytic rate constant (*k*_cat_) of the CYP154C3s toward the substrates were calculated and the results are summarized in Table 2 and Fig. S3. Both CYP154C3s exhibited broad selectivity towards various steroids. With increases in the substrate concentrations up to 500 μM, the progress of the reaction was investigated. With increases in the substrate concentration, the reaction rates decreased slowly after reaching a maximum rate. Both CYPs showed the maximum activity of substrate **9** with *K*_m_ values of 6.230 ± 0.710 and 10.670 ± 1.220 μM product/μM CYP and turnover numbers of 49.980 ± 1.260 and 45.900 ± 1.740 μM product/μM CYP for CYP154C3-1 and CYP154C3-2, respectively (Fig. 5). For most of the substrates, CYP154C3-1 demonstrated slightly higher activity compared to CYP154C3-2.

Coupling efficiencies are essential parameters for examining the efficiency of the catalytic system as a function of the used substrate. The coupling efficiency represents the percentage of NADH oxidized, which, in this case, was used for steroid hydroxylation. The coupling efficiencies of the substrates were calculated and the values were in the range of 8–39% and remained similar between the two CYP154C3s (Table 1). The coupling efficiency of **9** was highest, with values of 39.490 ± 4.120% and 36.060 ± 4.280% for CYP154C3-1 and CYP154C3-2, respectively, showing a higher coupling efficiency of CYP154C3-1 than that of CYP154C3-2. Moreover, the calculated overall coupling efficiency for both enzymes was lower. The lower coupling efficiency revealed a significant loss of electrons from the cofactor NADH. This may be due to the heterologous redox partners used during the catalysis by CYP154C3s. The P450cam with Pdx and PdR displayed a coupling efficiency of 100% in the conversion of the substrate D-camphor [46]. This indicates that the coupling efficiencies were affected by the efficiency of the electron transfer from NADH to CYP heme through the redox partners, which ultimately depended upon the contact between the redox partners. To attain higher coupling efficiency, the interaction with the redox partner protein should be optimal.

### In Vitro Study of Steroid Hydroxylation

**Fig. 6:**
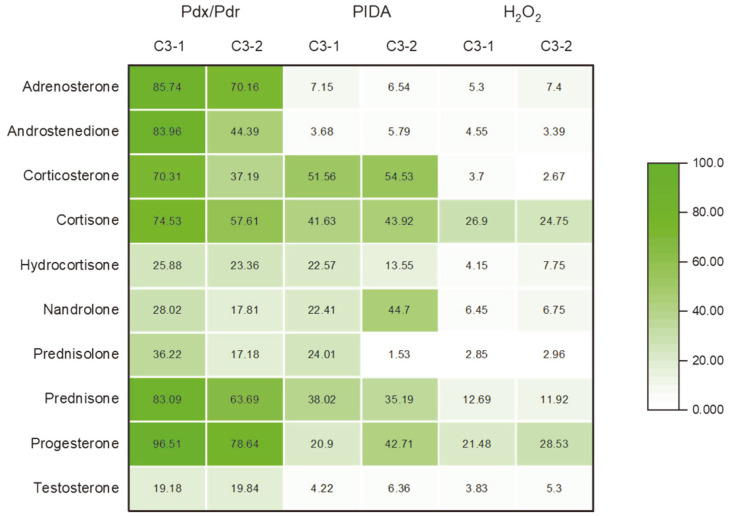
Heat map of the total product formation using different redox systems (A, CYP: Pdx: Pdr at a ratio of 1: 8: 2; B, 3 mM PIDA; and C, 50 mM H_2_O_2_) with purified CYP154C3s. The reaction time was 2 h at 30°C. The percentage turnover was derived from the ratio of the product peak area to the sum of the substrate and product area on H PLC analysis. C3-1 and C3-2 represent CYP154C3-1 and CYP154C3-2, respectively. The number inside the box represents the percentage of products formed. The intensity of the color indicates the different substrate conversion ratios.

The activity and specificity of the recombinant CYP154C3s were evaluated by performing CYP-dependent substrate conversion assays using a panel of substrates and by subsequent HPLC and LC-MS analyses of the hydroxylated products (Table S1). The in vitro reactions catalyzed by CYP154C3s were conducted separately in three different reaction systems consisting of NADH, H_2_O_2_, and PIDA. The total conversion of the substrate by the catalytic activity of the enzymes using different reaction systems is shown in the heatmap in Fig. 6.

In the presence of NADH cofactor, the purified heterologous redox partners Pdx-PdR were able to transfer electrons to the CYP154C3s. The in vitro conversion of substrates showed hydroxylation in more than one position, displaying no stereo- or regioselectivity (Fig. S4). LC-MS analysis of the reaction mixtures of **1, 2, 6**, and **10** revealed single monohydroxylated peaks (Fig. S5). Analysis of **3, 4, 7**, and **9** showed two monohydroxylated peaks, with **4** and **7** having additional di-hydroxylated peaks and **9** having two di-hydroxylated peaks. Three monohydroxylated peaks were observed for **5** and **8**, with an additional di hydroxylated peak for **8**. The substrates hydroxylated at single and multiple positions showed major structural differences. The finding showed that substrates with a functional group attached to the C17 position (**1, 2, 6**, and **10**) produced a single monohydroxylated product, whereas substrates with a functional group at the C21 and C11 positions like **3, 4, 5, 7**, and **8** generated multiple hydroxylated products. Therefore, it can be inferred that the C17 position functional group did not affect the product formation pattern, since **3**, without a C17 functional group was hydroxylated to multiple monohydroxylated products. This suggests that the functional group present at the C11 and C21 positions played a significant role in steroid hydroxylation by the CYP154C3s. A study of the substrate-bound crystal structure of CYP154C5 showed a major difference in the orientation of steroid-binding between C-17-hydroxyl/carbonyl-substituted steroids and C17-acetyl-substituted steroids [41]. A previous study reported that CYP154C8 had higher selectivity for the substrates with hydroxyl or keto groups at the C17 position compared to substrates with bulkier C17 substituents [17].

The product analysis of a single monohydroxylated peak revealed possible hydroxylation at the C16 position. The presence of an ion mass of m/z 121 and 145 of the unmodified steroidal A/B ring indicates possible C16 hydroxylation [47, 48]. The single monohydroxylated peaks (P1) of **2** and **10** were identified as 16α-hydroxyandrostenedione and 16α-hydroxytestosterone based on coelution with a standard (Fig. S5). The major product of **9** was identified as 16α-hydroxyprogesterone. The major monohydroxylated product (P1) of **3** was identified as 21α-hydroxycorticosterone by coelution with a standard and the other monohydroxylated product (P2) was a possible C16-hydroxylated product by comparison of the HPLC retention time. Due to the low conversion of the products, structural elucidation of the products by nuclear magnetic resonance (NMR) could not be achieved.

The conversion of the substrate by an alternate redox partner was also investigated by conducting an in vitro assay supported by the oxygen surrogates H_2_O_2_ and PIDA. The bacterial peroxygenases (CYP152 family) use H_2_O_2_ in the peroxide shunt pathway to catalyze reactions [8]. When using H_2_O_2_ in an in vitro assay, the oxidative degradation of heme by H_2_O_2_ is a major issue. Therefore, the H_2_O_2_ tolerance test was performed to determine the effect of H_2_O_2_ on the CYP154C3-mediated reaction. The test was performed using a range of H_2_O_2_ (0.2–200 mM) and observing the decrease in the Soret absorbance of the oxidized form of the CYP154C3s. Both CYP154C3s were active in high H_2_O_2_ concentrations (>10 mM). The optimal conversion of the substrates occurred at ~65 mM H_2_O_2_ and 3 mM PIDA (data not shown). Similar results for the optimal H_2_O_2_ and PIDA concentrations for CYP154C4 and CYP154C8 were reported [17, 25]. Additionally, it was also reported that the choice of suitable surrogate redox partners as well as reducing equivalents played an important role in the product distribution and catalytic efficiency of CYP enzymes [9].

The activity of CYP154C3s in the presence of H_2_O_2_ with steroid substrates was low with little change in the product distribution pattern (Fig. S4). For **4**, the use of H_2_O_2_ significantly increased the dihydroxylated product formed, surpassing the product formed using Pdx/PdR and PIDA (Fig. S5D). A single monohydroxylated product and two mono and one dihydroxylated product were observed with **1** and **7**, respectively, using Pdx/PdR (Figs. S5A and S5G). However, no product formation was observed using H_2_O_2_. For all of the other substrates, very little product formation was observed using H_2_O_2_.

The catalytic conversion of the substrates using PIDA was relatively low compared to Pdx/PdR but higher compared to H_2_O_2_ (Fig. S4). New products were observed with **6** when PIDA was used (Fig. S5F). LC-MS analysis showed two additional monohydroxylated products of **6** for both CYPs. The use of PIDA for both the CYP154C3s enhanced the catalytic conversion of **3** (Fig. S5C). The product distribution of **3** using PIDA remained unchanged but the product formation increased compared to Pdx/PdR. Among the two CYPs, the conversion rate of CYP154C3-2 for PIDA was comparatively high compared to CYP154C3-1. Very few CYPs have shown activity in the presence of PIDA. CYP2B1, CYP3A4, CYP121, CYP101A1, CYP106A2, and CYP154C8 were mammalian and bacterial CYPs with catalytic activity in the presence of iodosobenzene [9, 4^9^-^53^]. PIDA and iodosobenzene are single oxygen atom-containing surrogate oxidants and might share a similar monooxygenation mechanism [54].

CYP154C3s hydroxylated **4** and **7** at two different positions and **5** and **8** at three different positions in a reaction supported by Pdx-PdR-NADH. Moreover, additional product peaks were detected in addition to the two/three mono/di-hydroxylated products. LC-MS analysis of the additional peaks of these four substrates **4, 5, 7**, and **8** showed C–C bond cleavage products (P3 of **4**, P5 of **5**, P4 of **7**, and P5 of **8**) (Fig. S5). The HPLC chromatogram of 8 supported by NADH revealed an exact mass of m/z^+^ [M+H]^+^ 299.1635, and this mass precisely resembled the mass of a prednisone C–C bond cleavage product at C17. The C–C bond cleavage product was characterized as 1-dehydroadrenosterone based on coelution with a standard (Fig. S5H). Similarly, HPLC chromatogram analysis of **4, 5**, and **7** also showed C–C bond cleavage products with exact masses of m/z^+^ [M+H]^+^ 301.1798, 303.1952, 283.1684, respectively. By comparison to the standard, the C–C bond cleavage product of **4** was identified as adrenosterone. A previous study reported C–C lytic product formation by CYP154C8. CYP154C8 utilized both H_2_O_2_ and PIDA and demonstrated the formation of a C–C bond cleavage product [36]. Both the CYP154C3s were able to catalyze the cleavage of the C–C bond of steroid substrates (**4, 7, 5**, and **8**) supported by Pdx-PdR-NADH. The PIDA-constituted reaction for both CYPs showed a C–C lytic product of **4** only. However, only CYP154C-1 was able to produce a C–C lytic product of **4** with H_2_O_2_. For the other substrates, C–C lytic products were not observed using H_2_O_2_ and PIDA.

CYP154C3 from *S. griseus* showed steroid hydroxylation at the 16α position [24]. The pattern of hydroxylation by the CYP154C3s in the present study was different than the already-characterized CYP154C3 as the CYP154C3s in the present study were able to hydroxylate at the C21 position of steroids in addition to the C16 position. The use of alternate redox partners for catalytic activity completely changed the product distribution pattern. However, the use of oxygen surrogates H_2_O_2_ and PIDA generally resulted in a lower conversion of the substrates compared to the heterologous redox partners Pdx-PdR. Since individual CYPs are highly specific for particular redox partners, it is often necessary to establish the native electron transfer chain for a given CYP for optimal activity. The identification of the physiological redox partners for CYP154C3 is expected to increase the overall activity.

## Conclusions

In summary, two CYPs from *Streptomyces* species were expressed and purified. The activities were reconstituted using universally applicable heterologous redox partners (Pdx and PdR) from *P. putida*. This enabled the biochemical characterization, including the substrate and product selectivity and reaction kinetics of CYP154C3. Since the study used heterologous redox partners and surrogate oxygen species, identification of the physiological electron transfer partners for the efficient activity must be considered. Engineering bacterial CYP to accommodate alternative substrates and finding their new functions has been an important goal. Both CYP154C3s were easy to over-express and purify and are highly soluble proteins. Thus, they can be suitable candidates for protein engineering to allow the production of modified steroids for different biological applications.

## Supplemental Materials



Supplementary data for this paper are available on-line only at http://jmb.or.kr.

## Figures and Tables

**Table 1:** The dissociation constant (*K*_d_) of the CYP154C3s for steroids.

SN	Substrate	*K*_d_ [μM]	

		CYP154C3-1	CYP154C3-2
1	Adrenosterone	0.318 ± 0.026	0.443 ± 0.035
2	Androstenedione	0.351 ± 0.033	0.475 ± 0.054
3	Corticosterone	14.060 ± 2.570	44.490 ± 3.970
4	Cortisone	50.730 ± 6.140	26.880 ± 3.510
5	Hydrocortisone	80.390 ± 15.850	107.280 ± 13.860
6	Nandrolone	13.530 ± 2.620	6.580 ± 0.840
7	Prednisolone	45.400 ± 4.030	84.480 ± 9.060
8	Prednisone	20.690 ± 2.640	7.600 ± 0.430
9	Progesterone	0.288 ± 0.026	0.409 ± 0.037
10	Testosterone	2.630 ± 0.280	5.670 ± 0.550

The peak and trough were observed at 390 and 420 nm, respectively, for various substrate concentrations. The peak-to-trough absorbance differences were plotted against the respective substrate concentrations for determining the *K*_d_ value using the equation A_obs_ = A_max_ (([S]+[E_t_]+K_D_) - (([S]+[E_t_]+K_D_)^2^ - (4[S][E_t_])^0.5^)/2[E_t_].

**Table 2:** Michaelis-Menten constant (*K*_m_), maximum velocity (*V*_max_), the catalytic rate constant (*k*_cat_), and the coupling efficiency using purified CYP154C3s, PdR, Pdx, and 10 different steroid substrates.

SN	Substrate	*K*_m_ [μM]		*V*_max_ [μM Product / μM CYP / min]		*K*_cat_ [1/S]		Coupling efficiency (%)	

		CYP154C3-1	CYP154C3-2	CYP154C3-1	CYP154C3-2	CYP154C3-1	CYP154C3-2	CYP154C3-1	CYP154C3-2
1	Adrenosterone	30.110 ± 1.711	27.850 ± 1.820	0.804 ± 0.013	0.793 ± 0.017	48.240 ± 0.780	47.580 ± 1.020	29.440 ± 3.770	31.800 ± 4.380
2	Androstenedione	38.480 ± 4.350	26.980 ± 1.540	0.844 ± 0.031	0.787 ± 0.014	50.640 ± 1.860	47.220 ± 0.840	27.430 ± 3.920	22.040 ± 3.830
3	Corticosterone	59.700 ± 6.350	81.170 ± 11.630	0.941 ± 0.031	0.923 ± 0.046	56.460 ± 2.100	55.380 ± 2.760	19.380 ± 3.280	19.880 ± 3.300
4	Cortisone	83.500 ± 6.670	108.510 ± 12.480	0.930 ± 0.027	0.979 ± 0.041	55.800 ± 1.620	58.740 ± 2.460	21.650 ± 3.390	23.410 ± 3.920
5	Hydrocortisone	141.420 ± 18.120	170.750 ± 22.780	0.231 ± 0.015	0.228 ± 0.015	13.860 ± 1.600	13.680 ± 1.900	13.320 ± 2.860	11.870 ± 3.060
6	Nandrolone	53.820 ± 7.170	49.830 ± 11.180	0.893 ± 0.043	0.821 ± 0.068	53.580 ± 2.280	49.260 ± 4.050	23.530 ± 3.470	24.990 ± 3.590
7	Prednisolone	246.540 ± 38.310	280.990 ± 37.510	0.294 ± 0.023	0.285 ± 0.020	17.640 ± 1.380	17.100 ± 1.200	8.770 ± 3.140	9.160 ± 2.720
8	Prednisone	NA	NA	NA	NA	NA	NA	25.130 ± 2.460	24.230 ± 3.750
9	Progesterone	6.320 ± 0.710	10.670 ± 1.220	0.833 ± 0.210	0.765 ± 0.029	49.980 ± 1.260	45.900 ± 1.740	39.490 ± 4.120	36.060 ± 4.280
10	Testosterone	10.380 ± 0.830	18.260 ± 3.220	0.933 ± 0.025	0.915 ± 0.052	55.980 ± 1.500	54.900 ± 3.120	35.440 ± 4.320	33.680 ± 4.170

The overall apparent kinetic parameters were determined with a CYP: Pdx: Pdr concentration ratio of 1: 8: 2 for the purified CYP154C3s toward 10 substrates. Coupling efficiency was calculated as the percentage of NADH used for the formation of product over the total NADH consumption. The NADH consumption rate was calculated after subtracting the respective background NADH consumption. The results represent the mean values of triplicate measurements.
