# Supplementary material for: Enzymatic Characterization and Comparison of Two Steroid Hydroxylases CYP154C3-1 and CYP154C3-2 from Streptomyces Species
Source: J Microbiol Biotechnol. 2021 Jan 4;31(3):464–74. doi: 10.4014/jmb.2010.10020 (PMC9705902; doi:10.4014/jmb.2010.10020)

J. Microbiol. Biotechnol.
https://doi.org/10.4014/jmb.2010.10020

Table S1. LC-MS of parent compounds and their hydroxylated products

Substrate	Parent m/z$^+$ [M+H]$^+$	Product m/z$^+$ [M+H]$^+$
Adrenosterone	301.1797	317.1741 (P1)
Androstenedione	287.1999	303.1958 (P1)
Corticosterone	347.2219	363.2174 (P1), 363.2168 (P2)
Cortisone	361.2014	377.1964 (P1), 377.1968 (P2), 393.1918 (P3), 301.1798 (P4)
Hydrocortisone	363.2188	379.2119 (P1), 379.2118 (P2), 379.2116 (P3), 303.1952 (P4)
Nandrolone	275.2007	291.1966 (P1), 291.1956 (P2), 291.1971 (P3)
Prednisolone	361.2014	377.1958 (P1), 377.1960 (P2), 283.1684 (P4)
Prednisone	359.1860	375.1812 (P1), 375.1810 (P2), 375.1805 (P3), 391.1746 (P4), 299.1635 (P5)
Progesterone	315.2310	331.2276 (P1), 347.2205 (P2) 331.2270 (P3),
Testosterone	289.2156	305.2109 (P1)

Fig. S1. SDS-PAGE and spectral analysis of purified His-tagged CYP154C3-1 and CYP154C3-2. (A) SDS-PAGE analysis of soluble protein fraction (S), 20 mM imidazole eluted fraction (a), 100 mM imidazole eluted fractions containing 100 mm-1 (b) and 100 mM-2 (c), and standard protein marker (M). (**B**) SDS-PAGE of purified protein, CYP154C3-1 (lane 1, 45.44 kDa), CYP154C3-2 (lane 2, 45.91 kDa), PDX (lane 3, 10 kDa), and PDR (lane 4, 58 kDa), where M is the standard protein marker. The size of the protein marker in kilodaltons (kDa) is also presented. The dithionite-reduced CO-bound form of CYP154C3-1 (**C**) and CYP154C3-2 (**D**). (**E**) Substrate-binding spectra of CYP154C3-1 (black) and CYP154C3-2 (red). Progesterone binding at the CYP active site was characterized by a high-spin shift with a maximum absorbance (Soret peak) observed at 390 nm.

Fig. S2. Dissociation constants (K_d) of Cyp154C3-1 and CYP154C3-2 for various steroids. The K_d was determined by plotting the peak-to-trough absorbance difference (Abs_{390} - Abs_{420}) against various substrate concentrations using the equation $A_{obs} = A_{max}$ (([S]+[E_t]+K_D) - (([S]+[E_t]+K_D)^2 - (4[S][E_t])$^{0.5}$)/2[E_t].

Fig. S3. Michaelis-Menten plot of the substrates. Reaction mixture consisting of CYP: Pdx: Pdr in a ratio of 1: 8: 2 in the presence of varied substrate concentrations (0 – 400 μM). The rate of the reaction was determined and plotted against the substrate concentration.

Fig. S4. The product distribution of CYP154C3-1 (**A**) and CYP154C3-2 (**B**) from the hydroxylation of various steroids containing adrenosterone (**1**), androstenedione (**2**),

corticosterone (**3**), cortisone (**4**), hydrocortisone (**5**), nandrolone (**6**), prednisolone (**7**), prednisone (**8**), progesterone (**9**), and testosterone (**10**). Product formation was determined with system A (consisting of CYP: Pdx: Pdr at a ratio of 1: 8: 2 for the purified CYP154C3s), system B (3 mM iodobenzene PIDA), and system C (50 mM H_2O_2) for the 10 substrates. M1, M2, and M3 represent the different mono-hydroxylated products. D1 and D2 represent different di-hydroxylated products. The products other than mono and di-hydroxylations are represented by O1, O2, and O3.

Fig. S5. HPLC chromatogram of adrenosterone (**A**), androstenedione (**B**), corticosterone (**C**), cortisone (**D**), hydrocortisone (**E**), nandrolone (**F**), prednisolone (**G**), prednisone (**H**), progesterone (**I**), and testosterone (**J**) catalyzed by CYP154C3-1 and CYP154C3-2 in the reaction system supported by Pdx/Pdr, PIDA, and H_2O_2. The substrate peak and the corresponding product peaks are indicated by P1/P2/P3/P4/P5. The chromatogram of the authentic standard is also shown for some products. The LC-MS spectra of the substrate and the respective products are also presented.

Fig. S1.

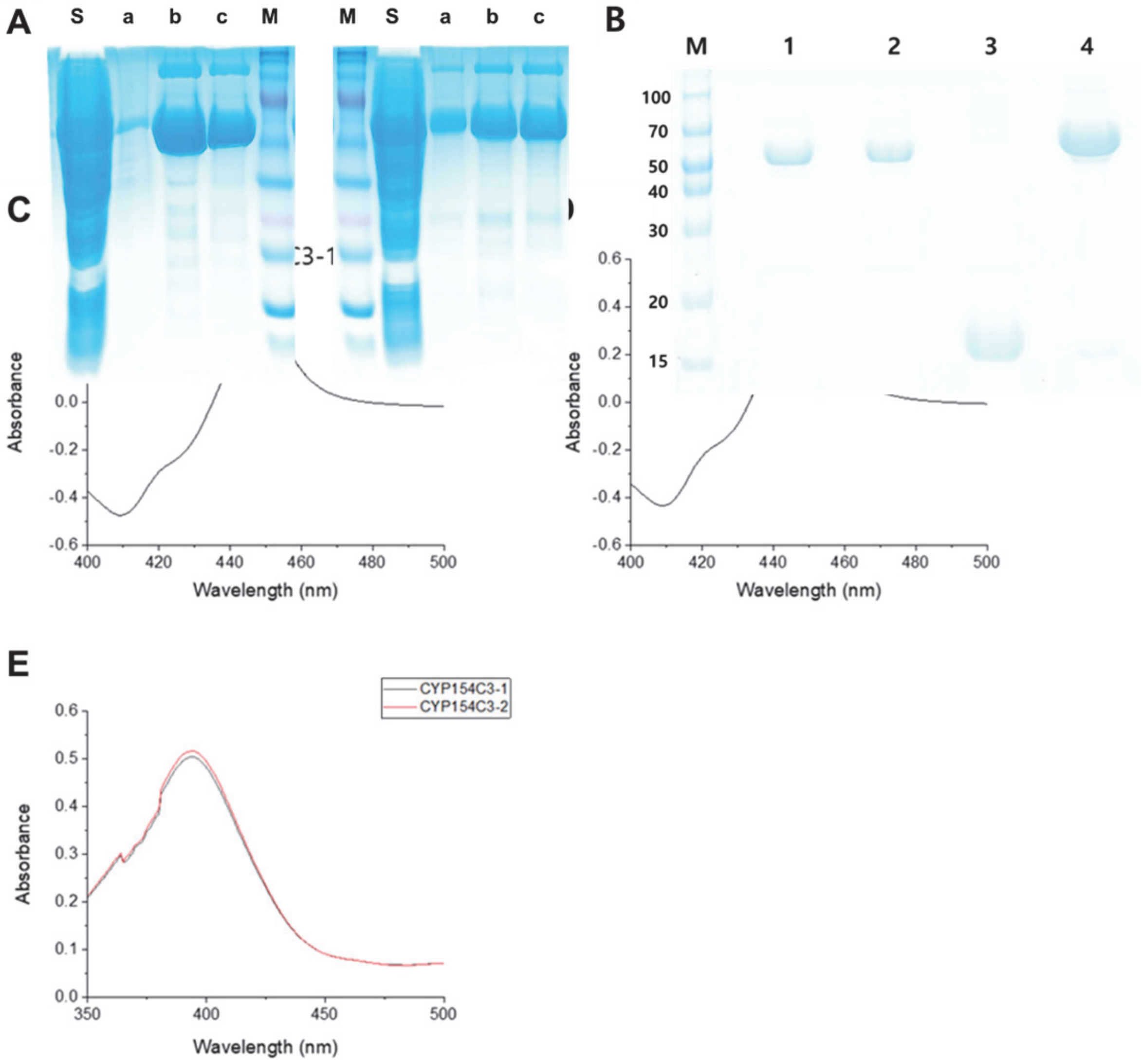
A
S a b c M
M S a b c
B
M 1 2 3 4
100
70
50
40
30
20
15
C
0.6
0.4
0.2
0.0
-0.2
-0.4
-0.6
Absorbance
400 420 440 460 480 500
Wavelength (nm)
E
CYP154C3-1
CYP154C3-2
0.6
0.5
0.4
0.3
0.2
0.1
0.0
Absorbance
350 400 450 500
Wavelength (nm)

Fig. S2.

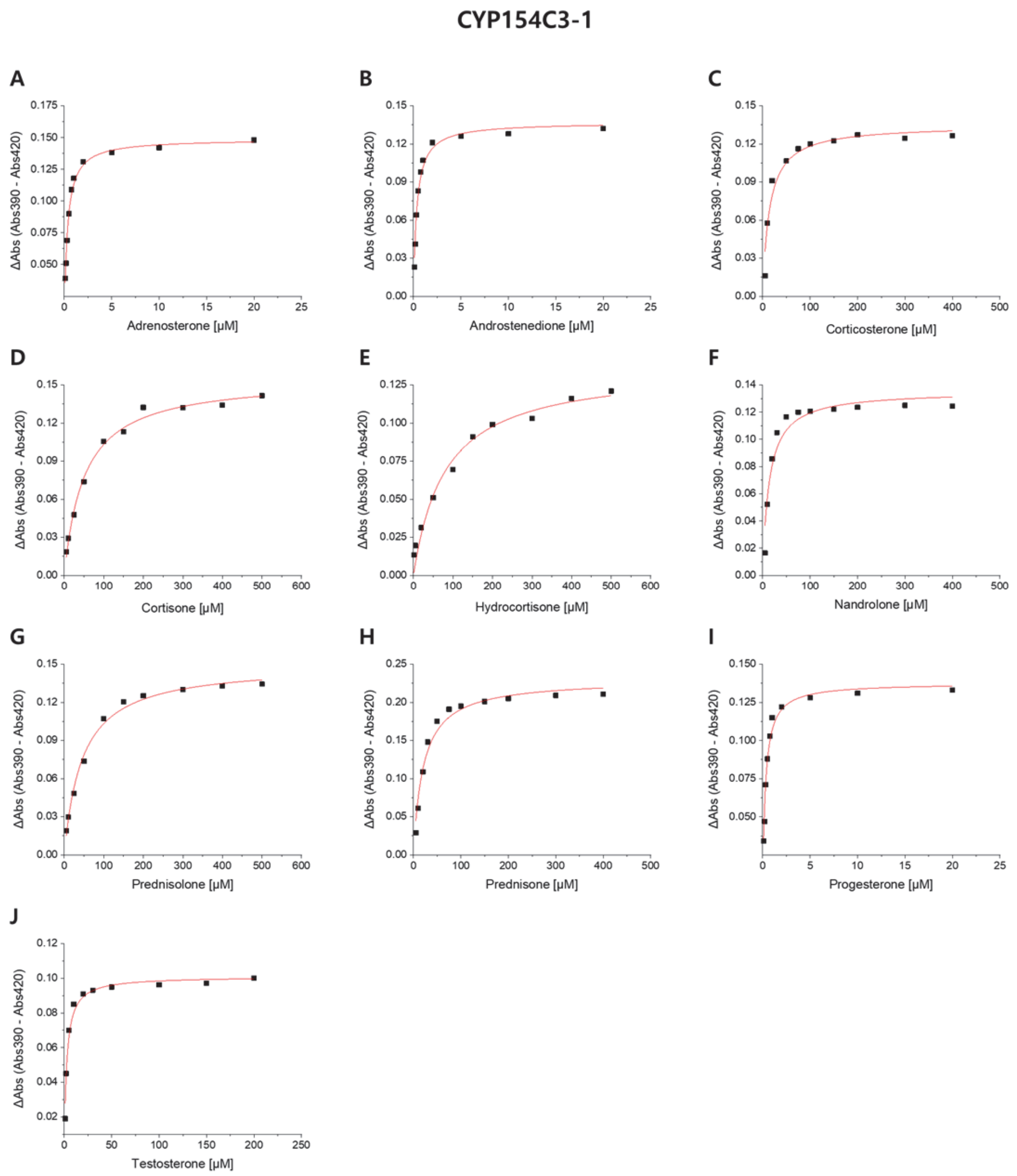
CYP154C3-1
A
ΔAbs (Abs390 - Abs420)
Adrenosterone [μM]
B
ΔAbs (Abs390 - Abs420)
Androstenedione [μM]
C
ΔAbs (Abs390 - Abs420)
Corticosterone [μM]
D
ΔAbs (Abs390 - Abs420)
Cortisone [μM]
E
ΔAbs (Abs390 - Abs420)
Hydrocortisone [μM]
F
ΔAbs (Abs390 - Abs420)
Nandrolone [μM]
G
ΔAbs (Abs390 - Abs420)
Prednisolone [μM]
H
ΔAbs (Abs390 - Abs420)
Prednisone [μM]
I
ΔAbs (Abs390 - Abs420)
Progesterone [μM]
J
ΔAbs (Abs390 - Abs420)
Testosterone [μM]

CYP154C3-2

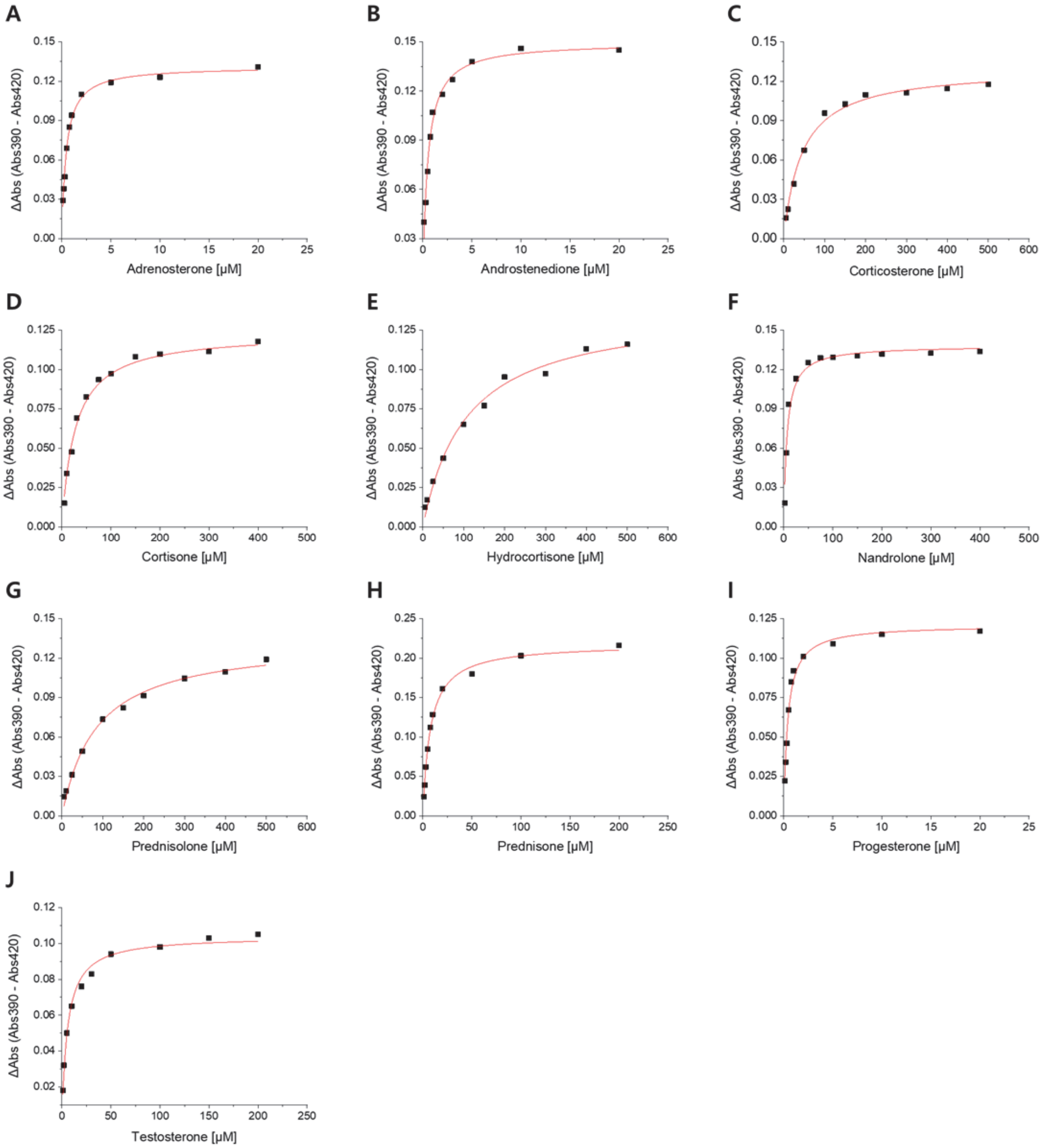
A
ΔAbs (Abs390 - Abs420)
Adrenosterone [µM]
B
ΔAbs (Abs390 - Abs420)
Androstenedione [µM]
C
ΔAbs (Abs390 - Abs420)
Corticosterone [µM]
D
ΔAbs (Abs390 - Abs420)
Cortisone [µM]
E
ΔAbs (Abs390 - Abs420)
Hydrocortisone [µM]
F
ΔAbs (Abs390 - Abs420)
Nandrolone [µM]
G
ΔAbs (Abs390 - Abs420)
Prednisolone [µM]
H
ΔAbs (Abs390 - Abs420)
Prednisone [µM]
I
ΔAbs (Abs390 - Abs420)
Progesterone [µM]
J
ΔAbs (Abs390 - Abs420)
Testosterone [µM]

Fig. S3.

CYP154C3-1

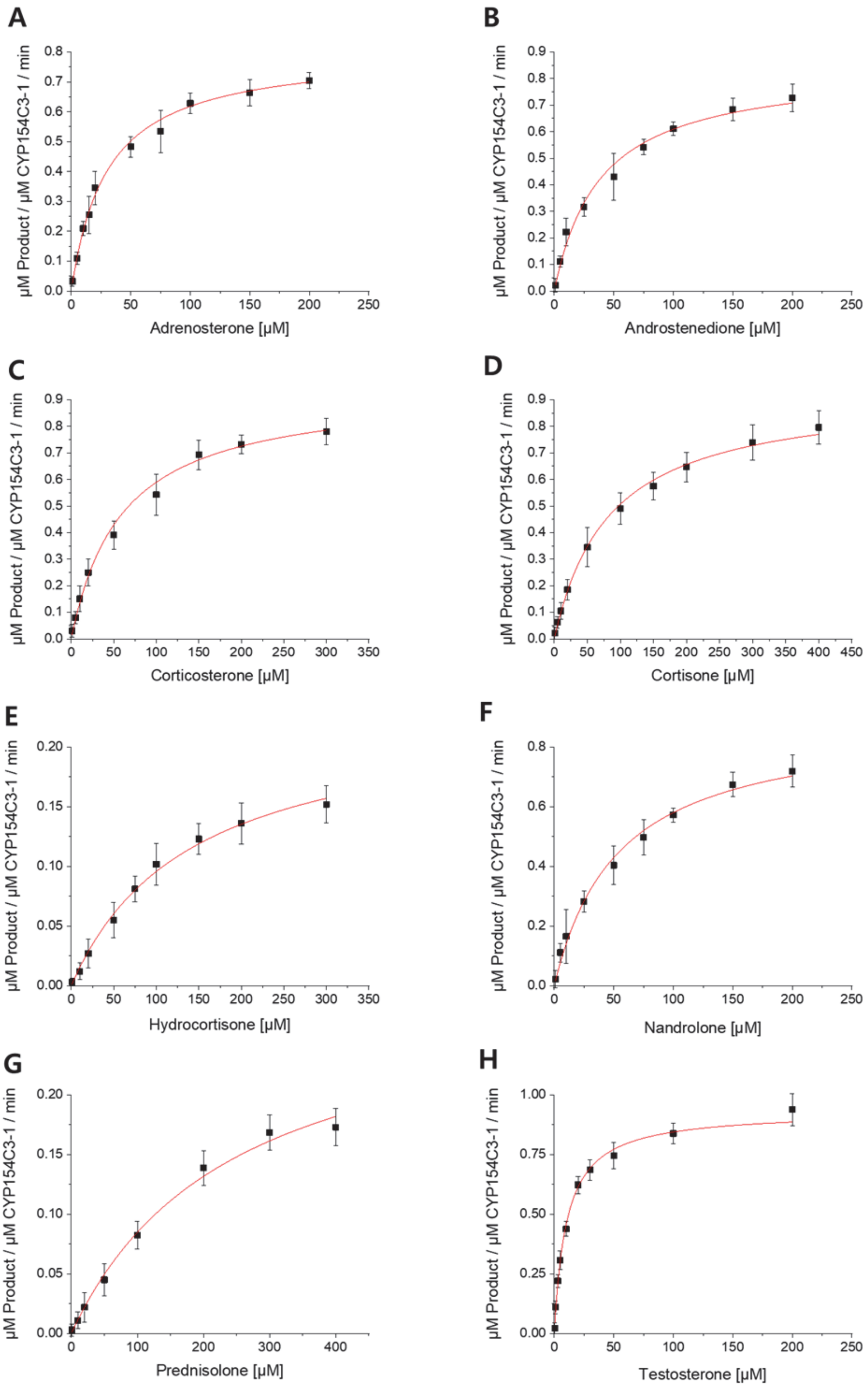

CYP154C3-2

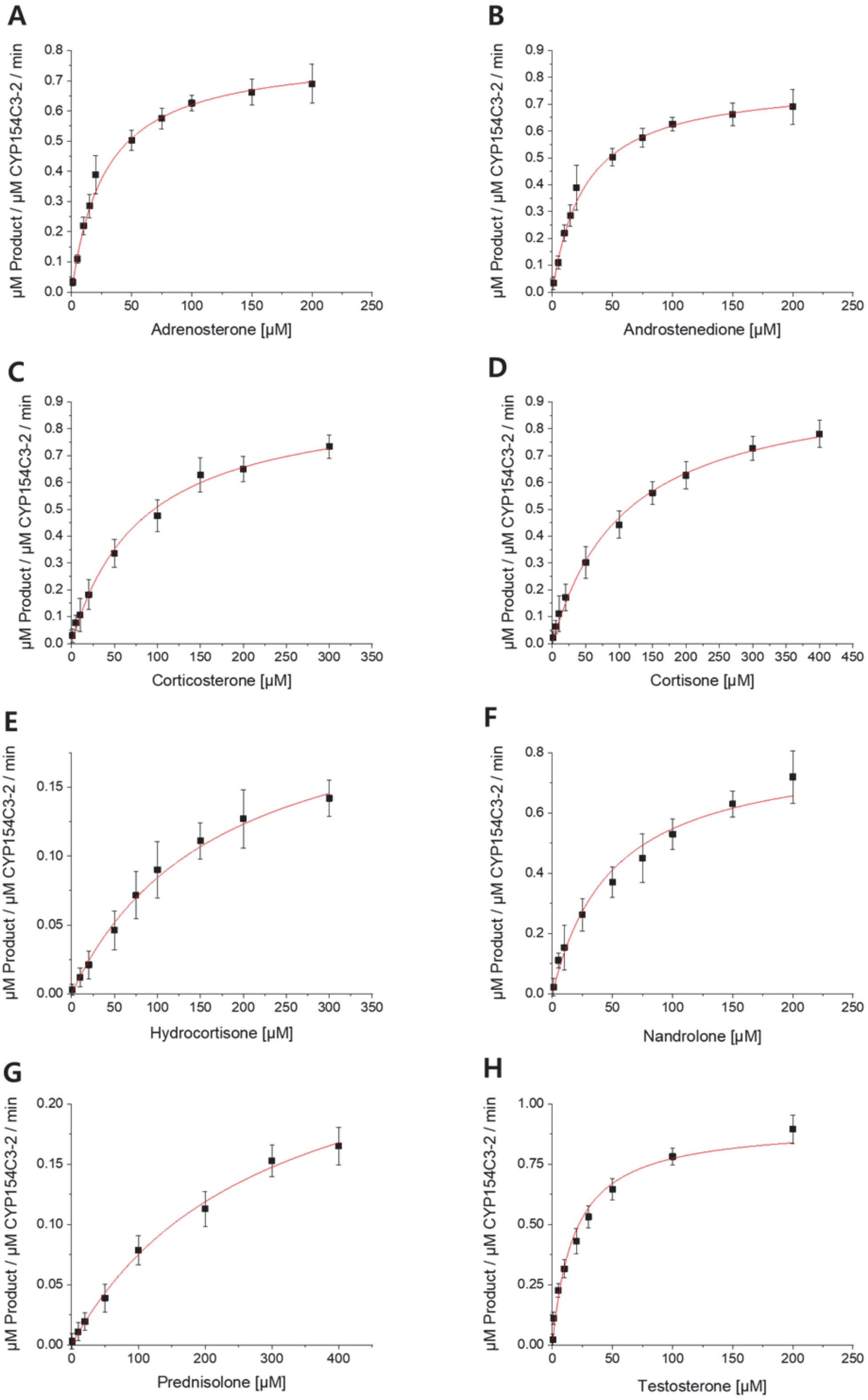
A
µM Product / µM CYP154C3-2 / min
Adrenosterone [µM]
B
µM Product / µM CYP154C3-2 / min
Androstenedione [µM]
C
µM Product / µM CYP154C3-2 / min
Corticosterone [µM]
D
µM Product / µM CYP154C3-2 / min
Cortisone [µM]
E
µM Product / µM CYP154C3-2 / min
Hydrocortisone [µM]
F
µM Product / µM CYP154C3-2 / min
Nandrolone [µM]
G
µM Product / µM CYP154C3-2 / min
Prednisolone [µM]
H
µM Product / µM CYP154C3-2 / min
Testosterone [µM]

Fig. S4.

A

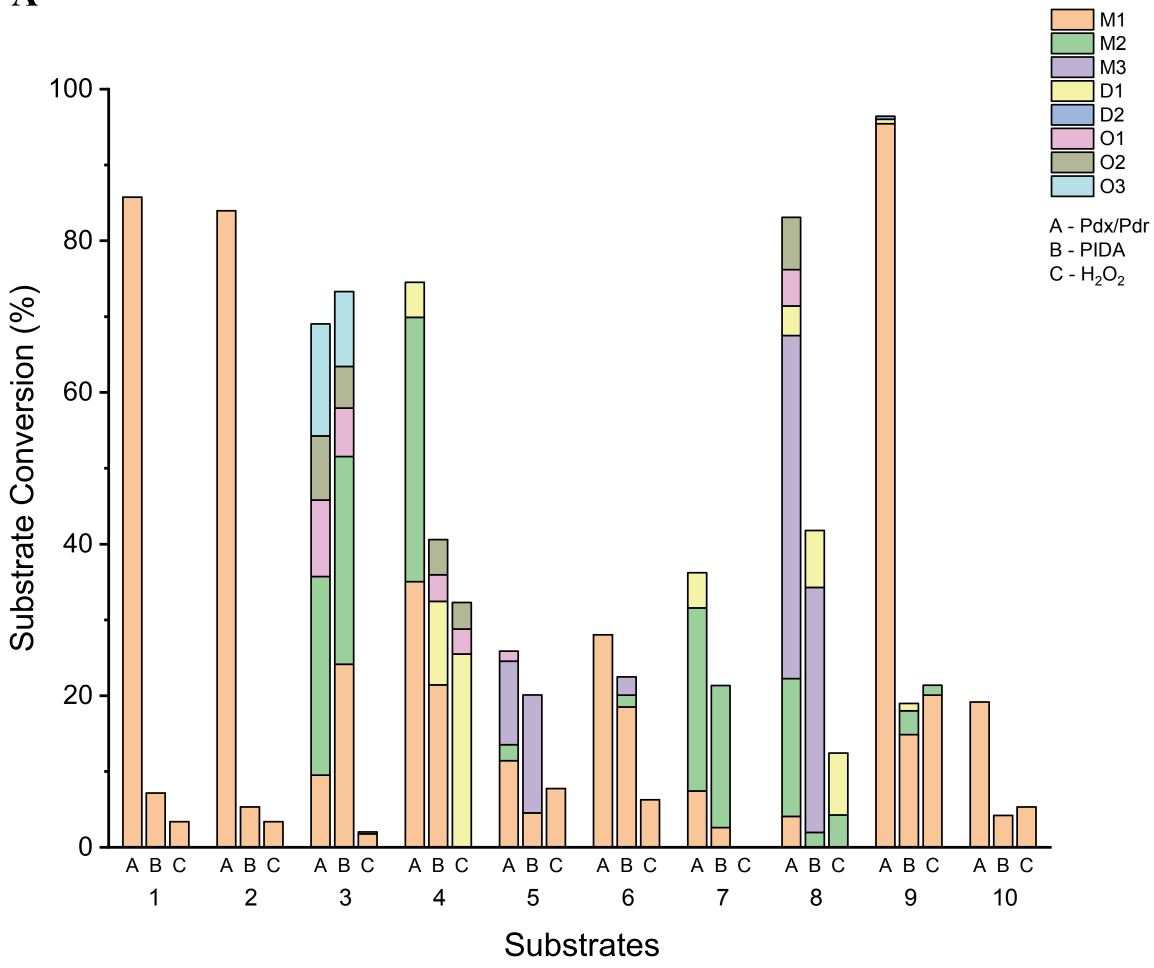

M1
M2
M3
D1
D2
O1
O2
O3
A - Pdx/Pdr
B - PIDA
C - H2O2
Substrate Conversion (%)
0
20
40
60
80
100
A B C
1
2
3
4
5
6
7
8
9
10
Substrates

B

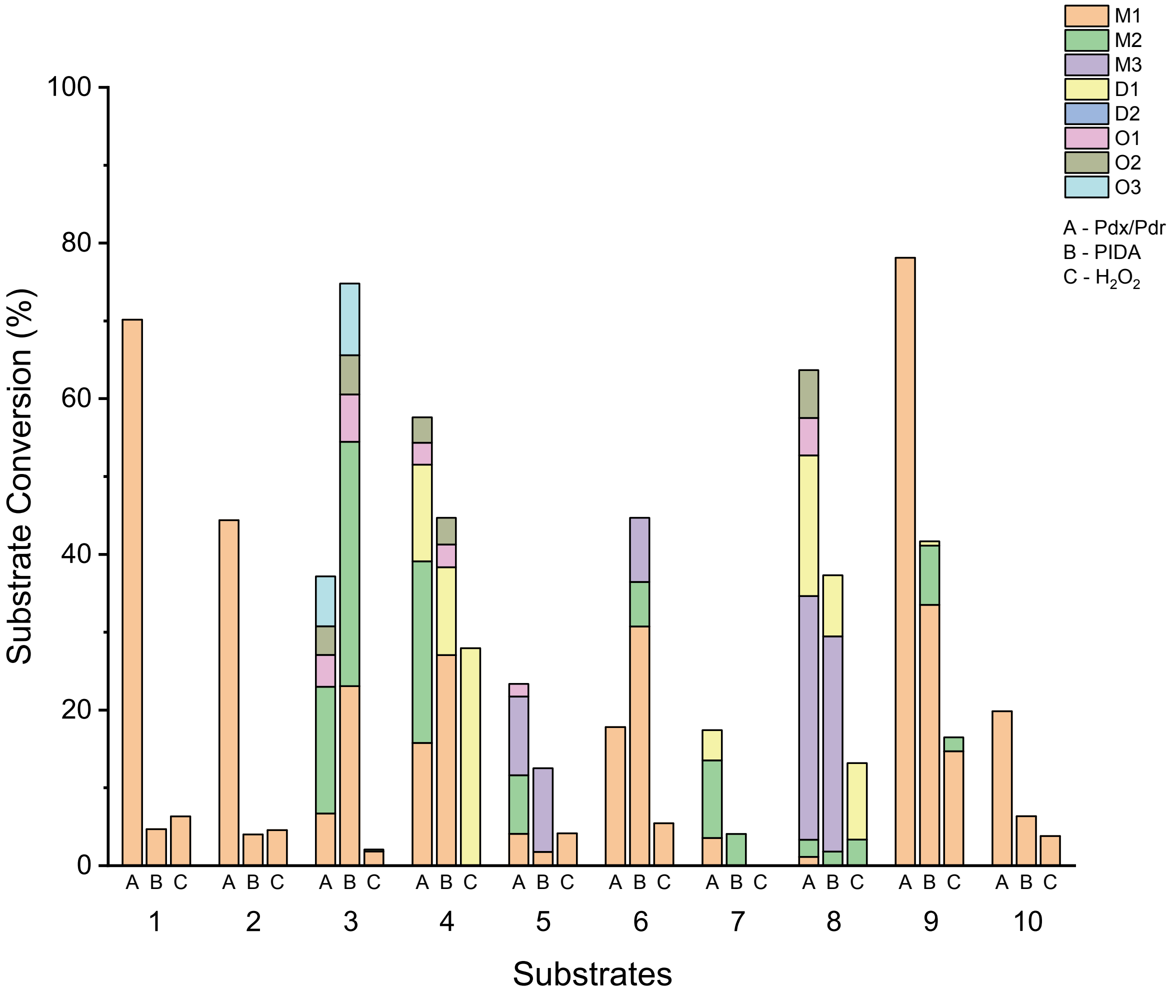
M1
M2
M3
D1
D2
O1
O2
O3
A - Pdx/Pdr
B - PIDA
C - H_2O_2
Substrate Conversion (%)
0
20
40
60
80
100
A B C
1
2
3
4
5
6
7
8
9
10
Substrates

Fig. S5.

A. Adrenosterone

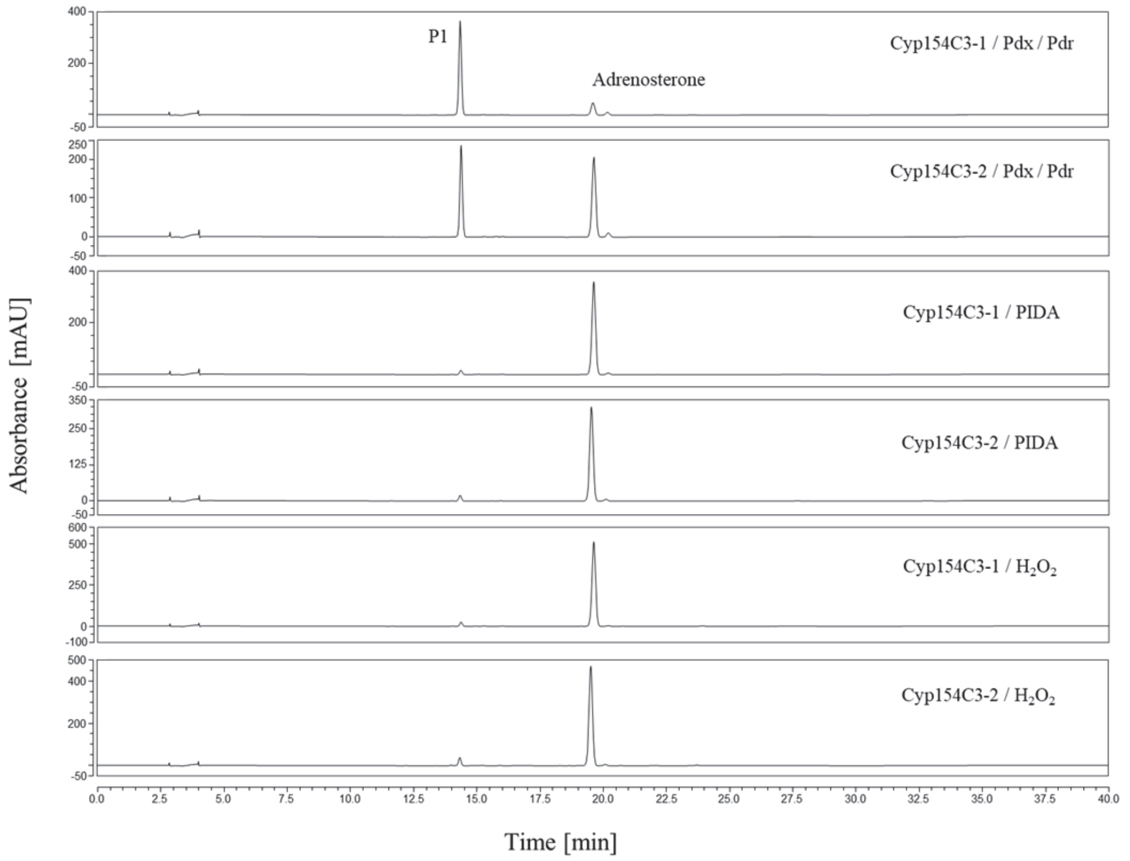

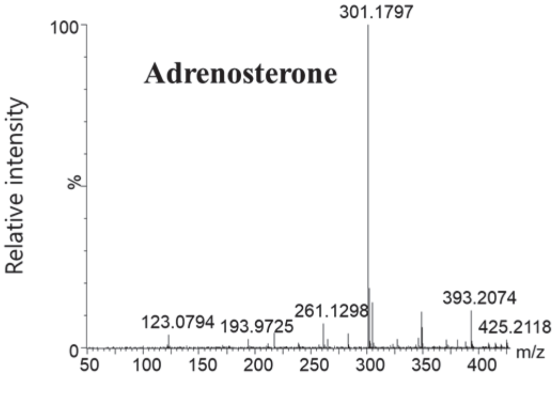

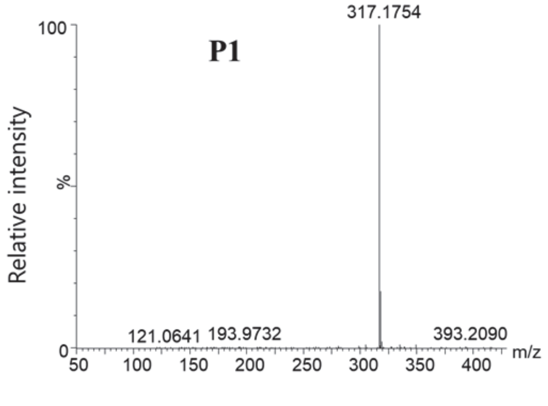

B. Androstenedione

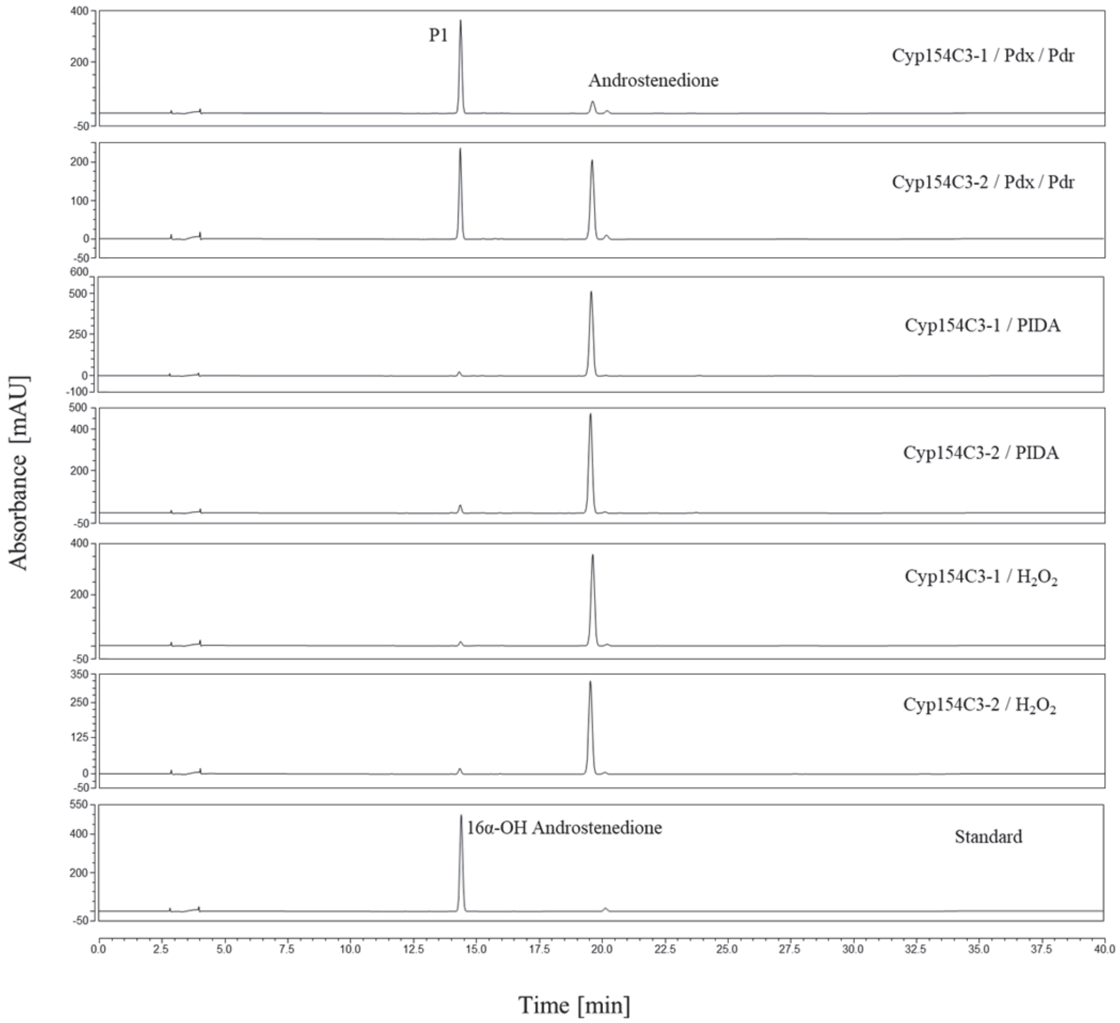

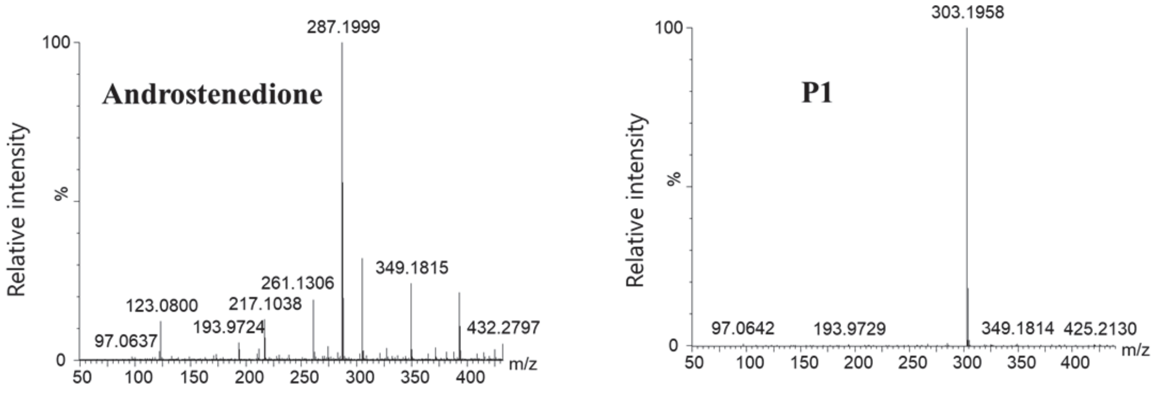

C. Corticosterone

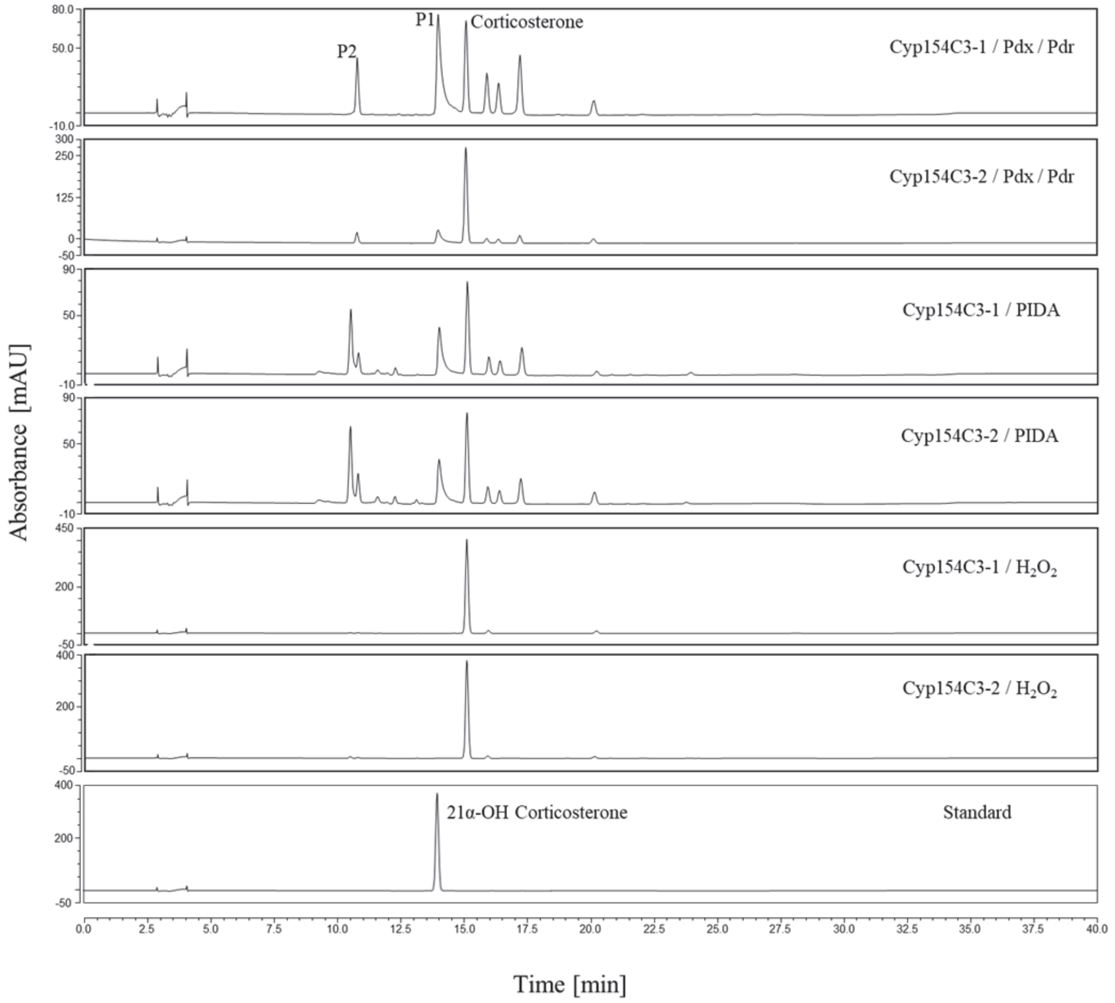

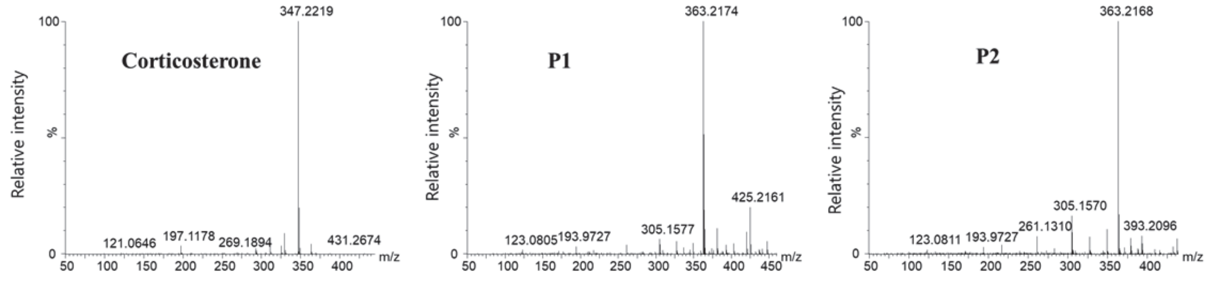

D. Cortisone

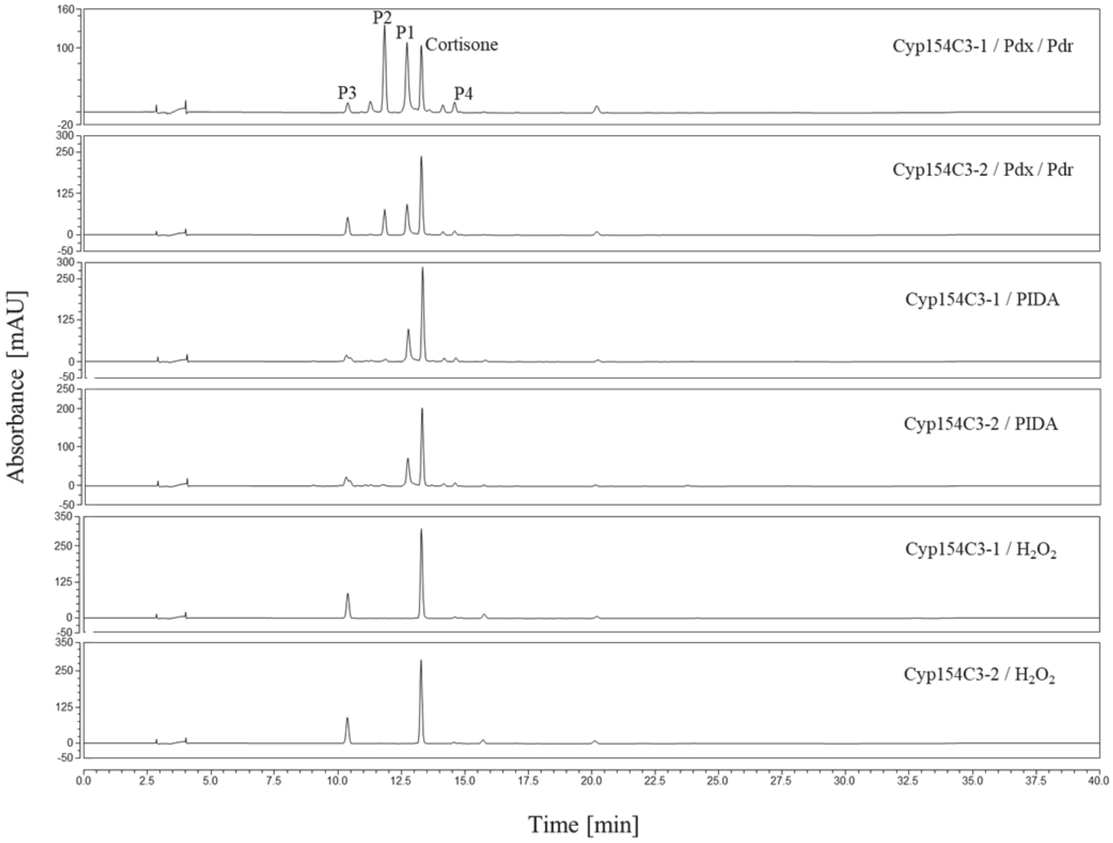

Absorbance [mAU]
Time [min]
P2
P1
Cortisone
P3
P4
Cyp154C3-1 / Pdx / Pdr
Cyp154C3-2 / Pdx / Pdr
Cyp154C3-1 / PIDA
Cyp154C3-2 / PIDA
Cyp154C3-1 / H_2O_2
Cyp154C3-2 / H_2O_2

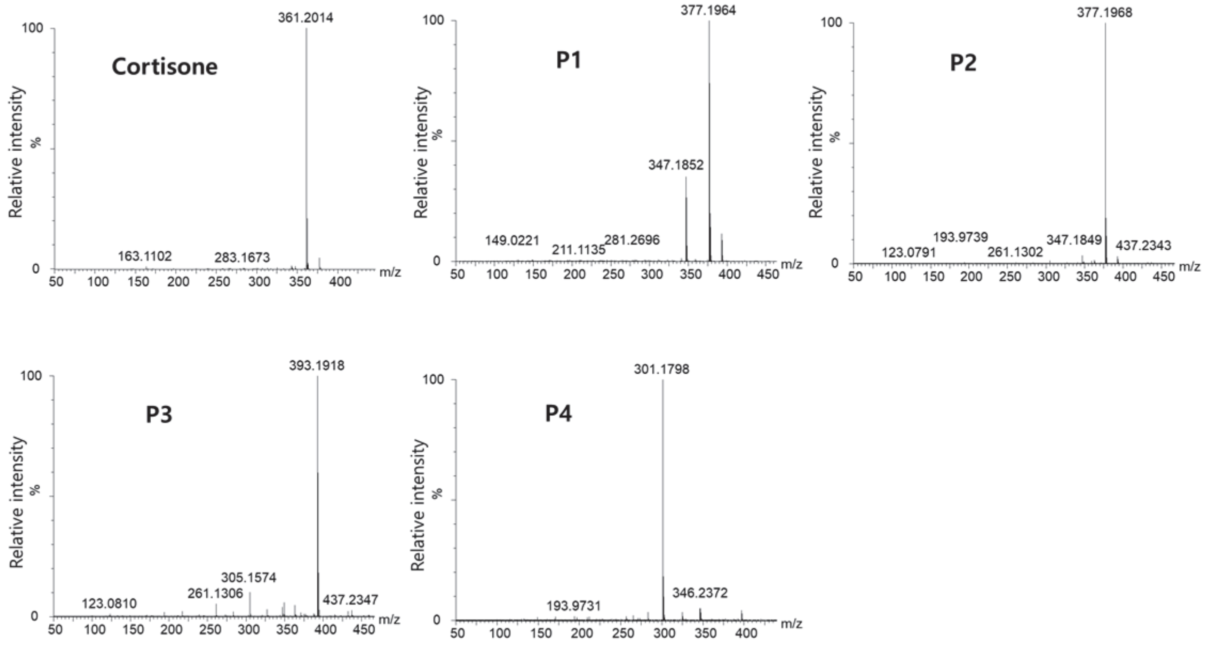

Cortisone
361.2014
163.1102
283.1673
P1
377.1964
347.1852
149.0221
211.1135
281.2696
P2
377.1968
193.9739
123.0791
261.1302
347.1849
437.2343
P3
393.1918
305.1574
261.1306
123.0810
437.2347
P4
301.1798
193.9731
346.2372
Relative intensity
%
m/z

E. Hydrocortisone

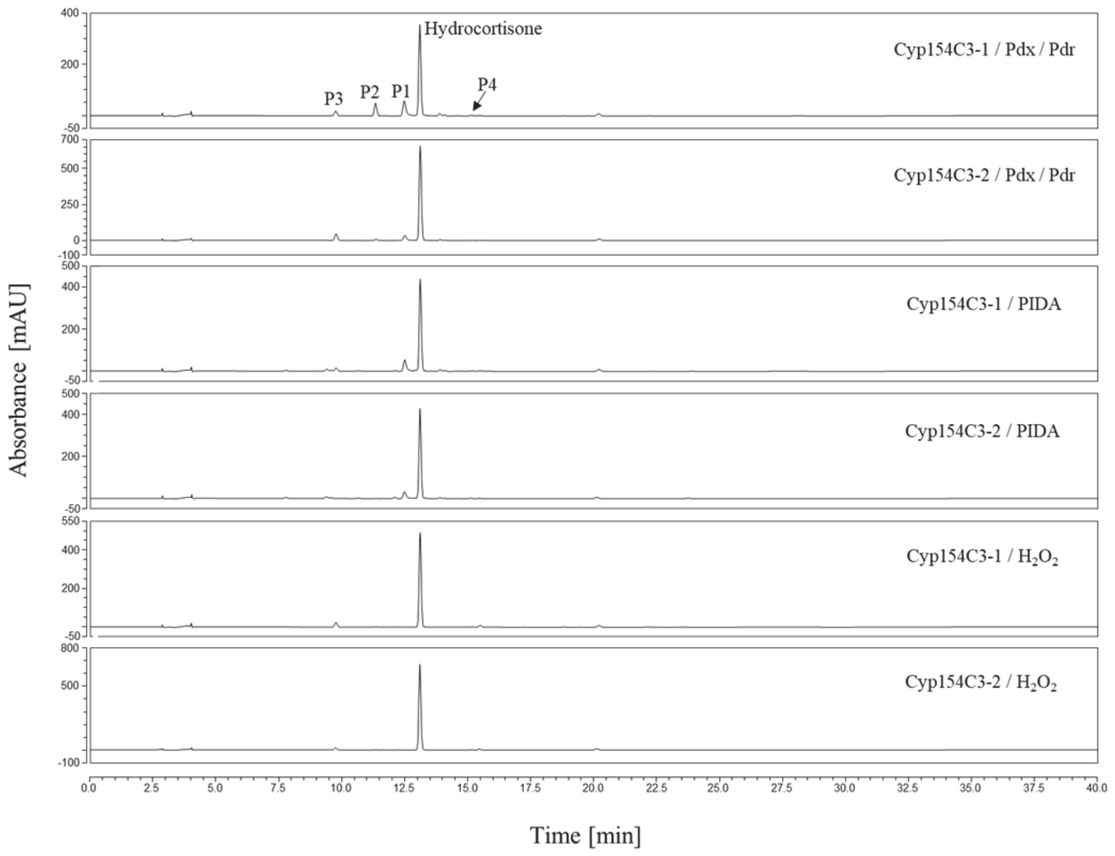

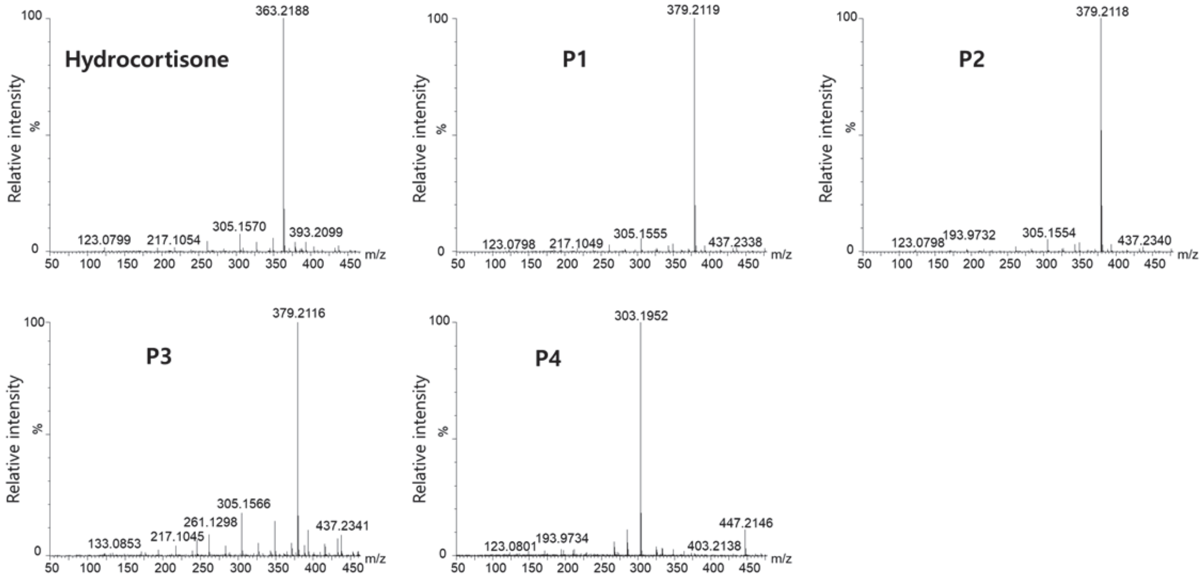

F. Nandrolone

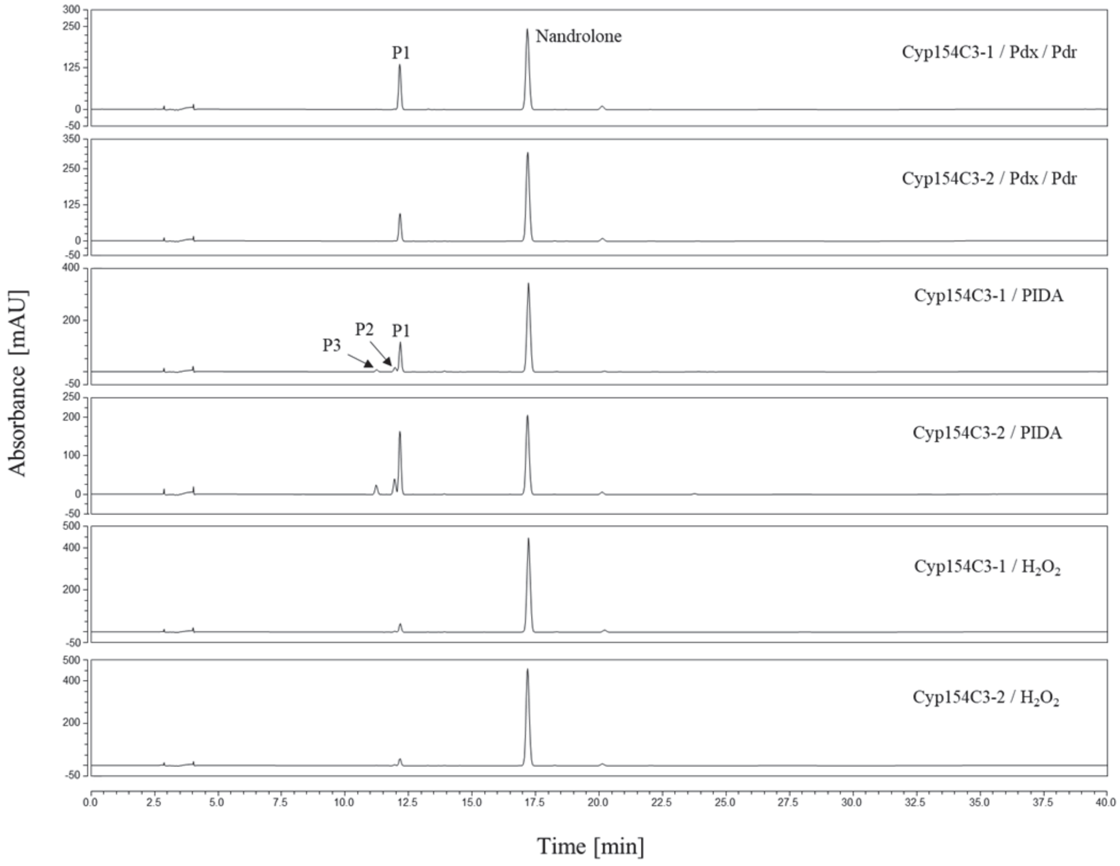

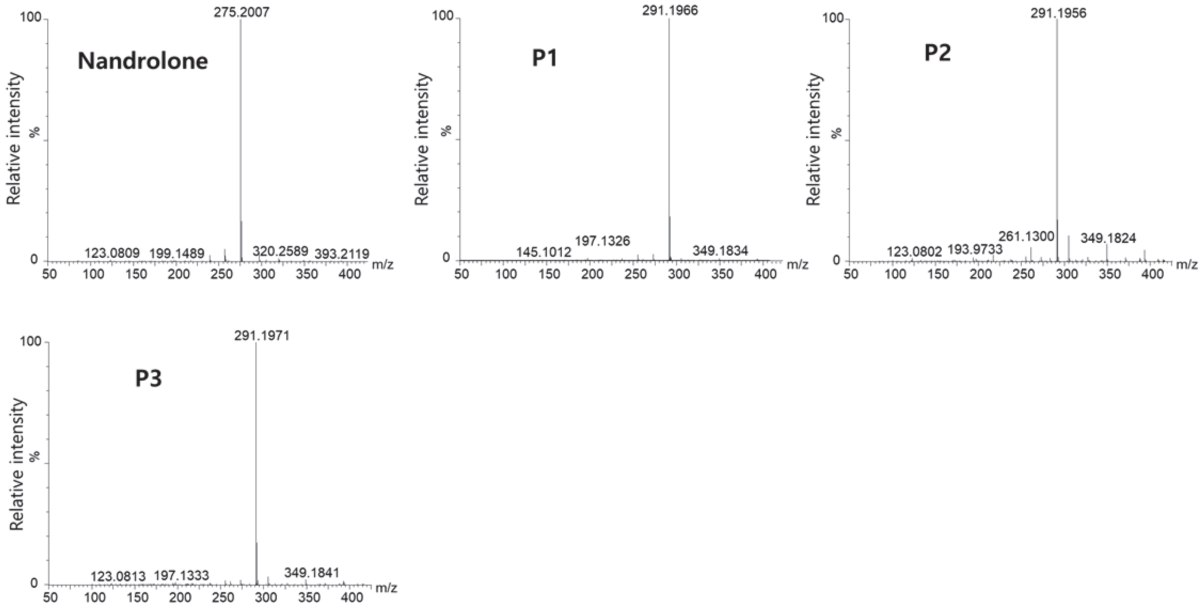
Nandrolone
275.2007
123.0809
199.1489
320.2589
393.2119
P1
291.1966
145.1012
197.1326
349.1834
P2
291.1956
123.0802
193.9733
261.1300
349.1824
P3
291.1971
123.0813
197.1333
349.1841
Relative intensity
%
m/z

G. Prednisolone

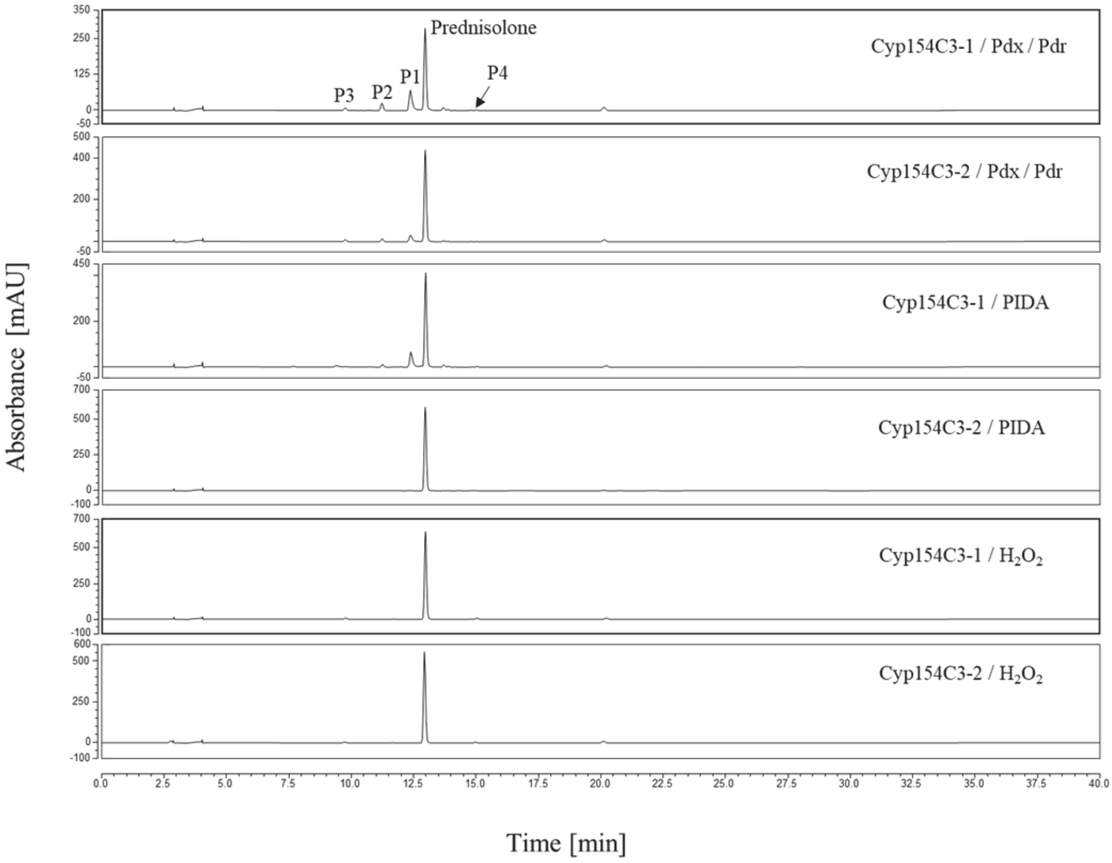

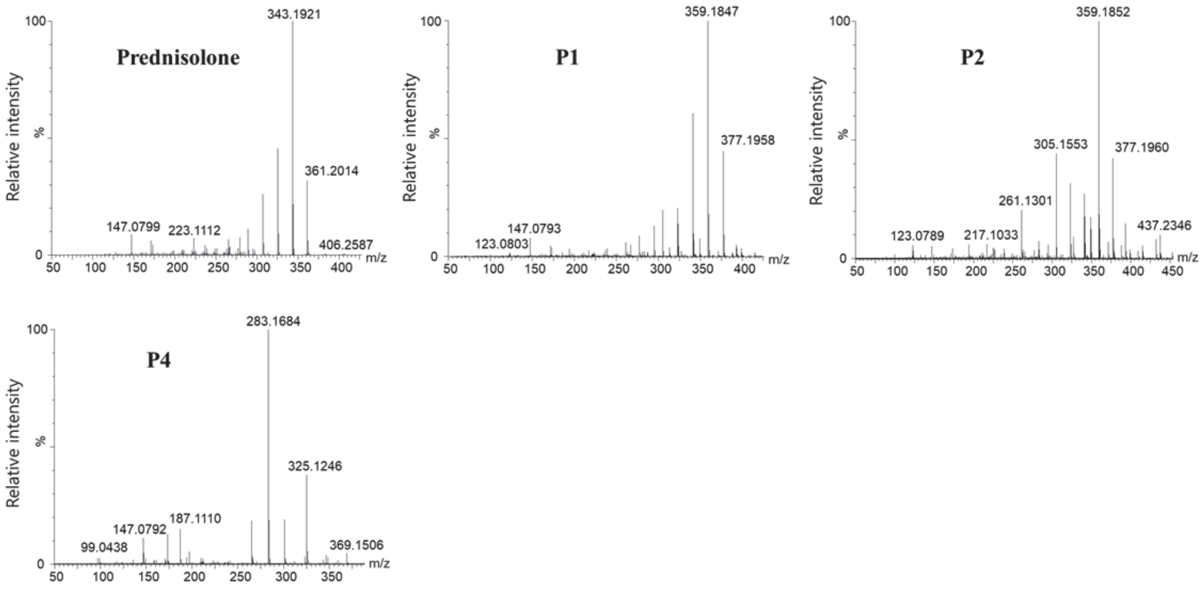

H. Prednisone

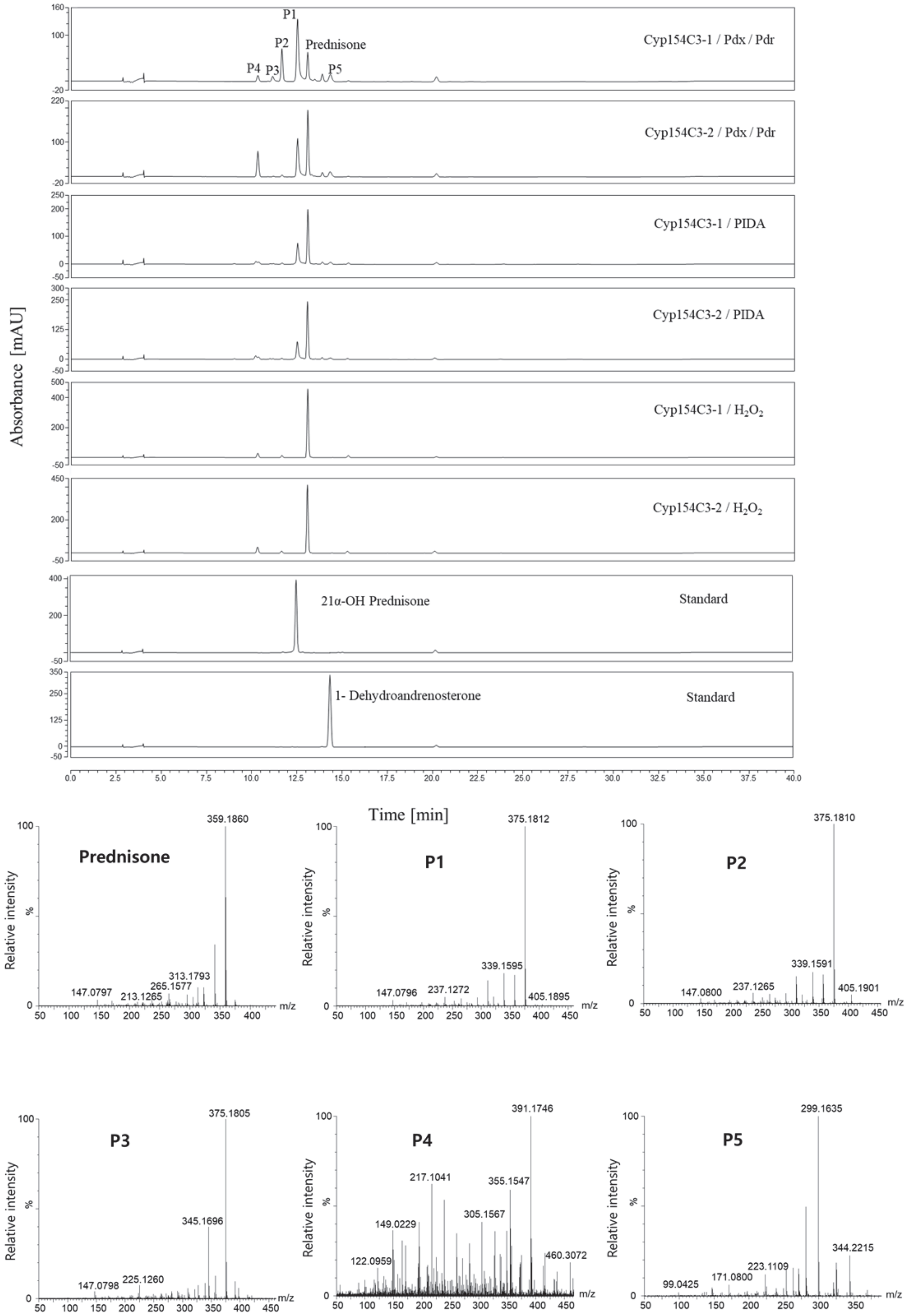

I. Progesterone

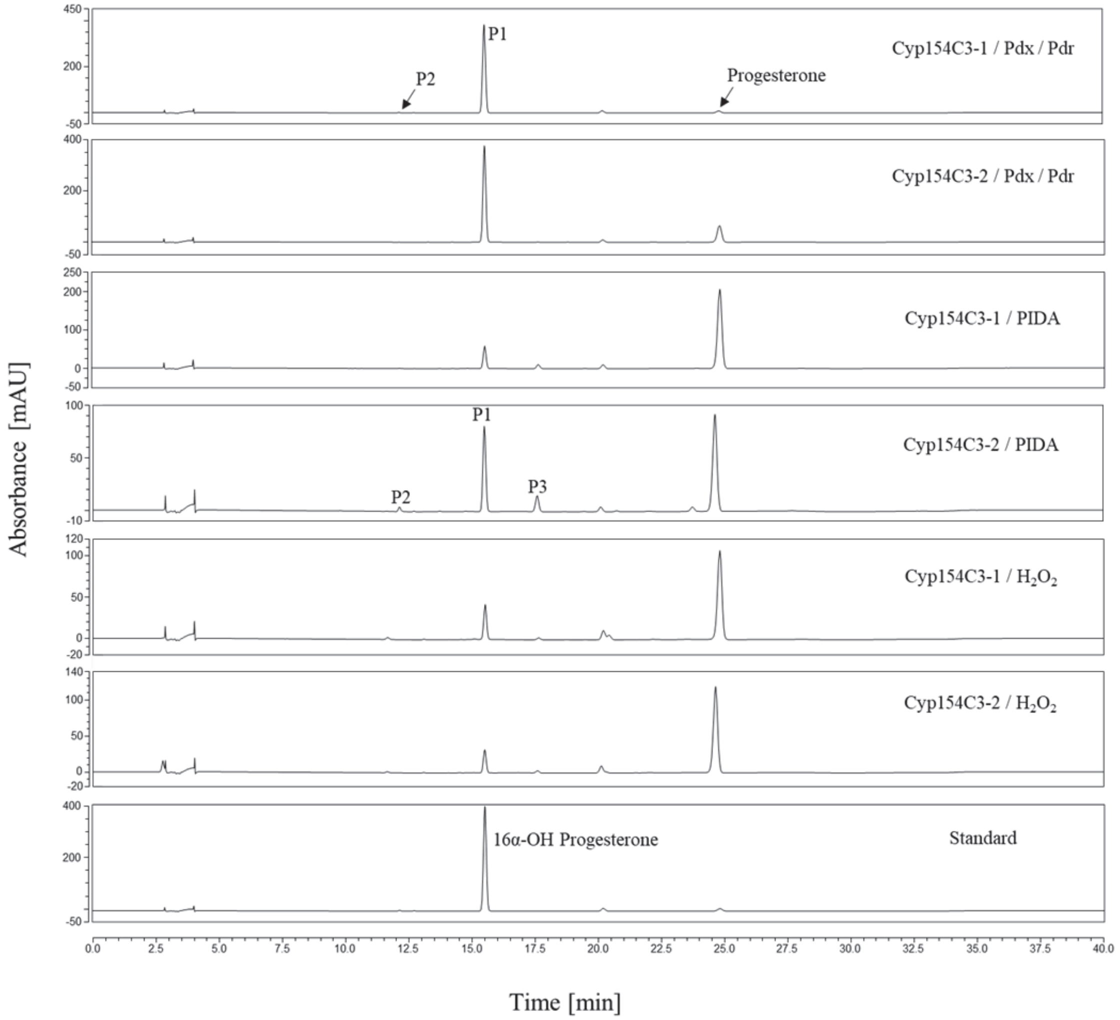

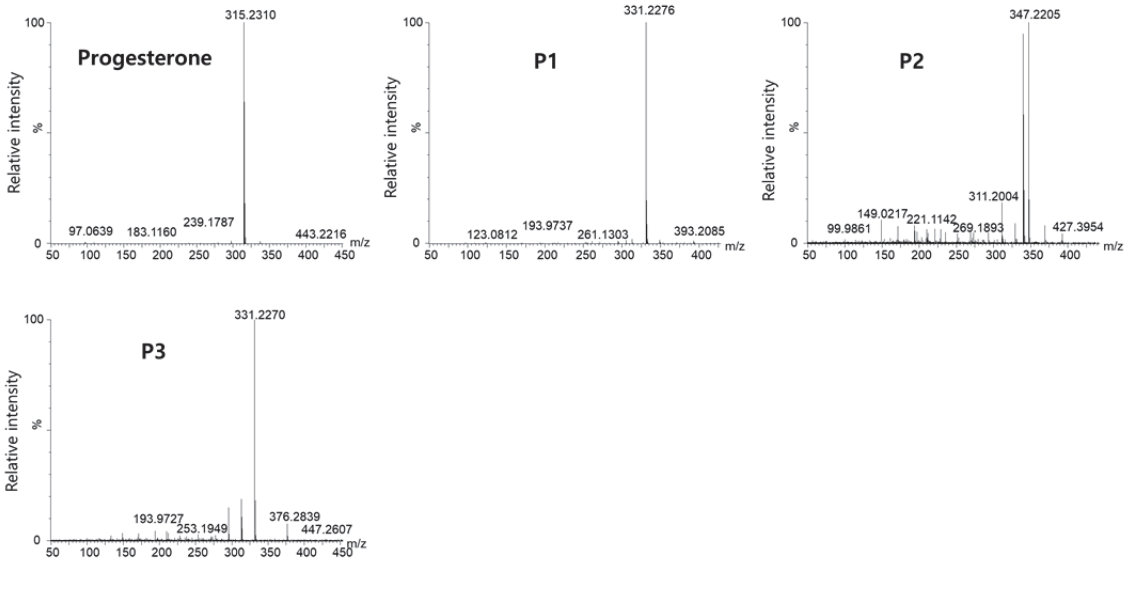

J. Testosterone

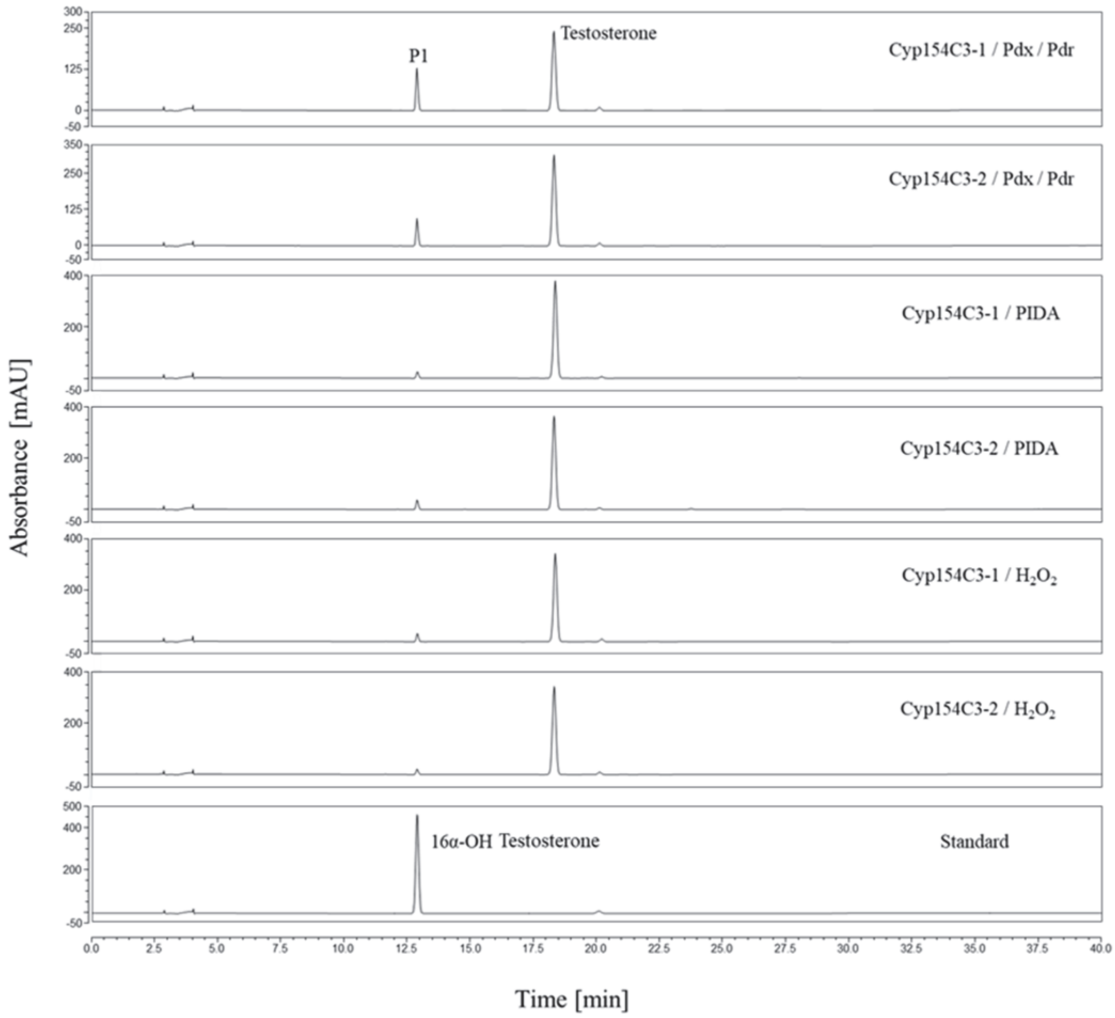

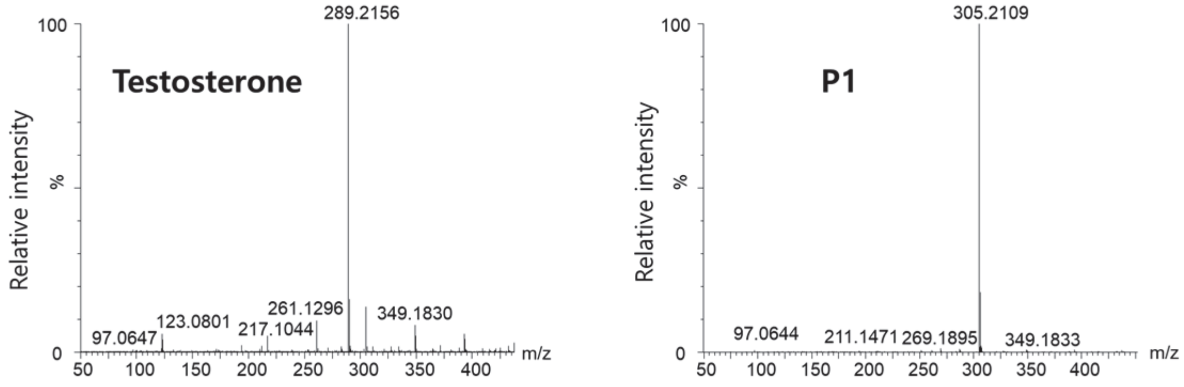

Supplement: Supplementary file 1 [file jmb-31-3-464-supple.pdf]
